# Adult mortality of diseases and injuries attributable to selected metabolic, lifestyle, environmental, and infectious risk factors in Taiwan: a comparative risk assessment

**DOI:** 10.1186/s12963-017-0134-4

**Published:** 2017-05-03

**Authors:** Wei-Cheng Lo, Chu-Chang Ku, Shu-Ti Chiou, Chang-Chuan Chan, Chi‐Ling Chen, Mei-Shu Lai, Hsien-Ho Lin

**Affiliations:** 1 Graduate Institute of Epidemiology and Preventive Medicine, College of Public Health, National Taiwan University, 17 Xuzhou Rd, Rm 706, Taipei, 10055 Taiwan; 2 Taiwan Cancer Registry, Taipei, Taiwan; 3 School of Health and Related Research (ScHARR), University of Sheffield, Sheffield, UK; 4 Health Promotion Administration, Ministry of Health and Welfare, Taipei, Taiwan; 5 Institute of Public Health, National Yang-Ming University, Taipei, Taiwan; 6 Institute of Occupational Medicine and Industrial Hygiene, College of Public Health, National Taiwan University, Taipei, Taiwan; 7 Global Health Center, College of Public Health, National Taiwan University, Taipei, Taiwan; 8 Graduate Institute of Clinical Medicine, Department of Internal Medicine and Hepatitis Research Center, National Taiwan University College of Medicine and Hospital, Taipei, Taiwan

**Keywords:** Burden of disease, Comparative risk assessment, Modifiable risk factors

## Abstract

**Background:**

To facilitate priority-setting in health policymaking, we compiled the best available information to estimate the adult mortality (>30 years) burden attributable to 13 metabolic, lifestyle, infectious, and environmental risk factors in Taiwan.

**Methods:**

We obtained data on risk factor exposure from nationally representative health surveys, cause-specific mortality from the National Death Registry, and relative risks from epidemiological studies and meta-analyses. We applied the comparative risk assessment framework to estimate mortality burden attributable to individual risk factors or risk factor clusters.

**Results:**

In 2009, high blood glucose accounted for 14,900 deaths (95% UI: 11,850–17,960), or 10.4% of all deaths in that year. It was followed by tobacco smoking (13,340 deaths, 95% UI: 10,330–16,450), high blood pressure (11,190 deaths, 95% UI: 8,190–14,190), ambient particulate matter pollution (8,600 deaths, 95% UI: 7,370–9,840), and dietary risks (high sodium intake and low intake of fruits and vegetables, 7,890 deaths, 95% UI: 5,970–9,810). Overweight-obesity and physical inactivity accounted for 7,620 deaths (95% UI: 6,040–9,190), and 7,400 deaths (95% UI: 6,670–8,130), respectively. The cardiometabolic risk factors of high blood pressure, high blood glucose, high cholesterol, and overweight-obesity jointly accounted for 12,120 deaths (95% UI: 11,220–13,020) from cardiovascular diseases. For domestic risk factors, infections from hepatitis B virus (HBV) and hepatitis C virus (HCV) were responsible for 6,300 deaths (95% UI: 5,610–6,980) and 3,170 deaths (95% UI: 1,860–4,490), respectively, and betel nut use was associated with 1,780 deaths from oral, laryngeal, and esophageal cancer (95% UI: 1,190–2,360). The leading risk factors for years of life lost were similar, but the impact of tobacco smoking and alcohol use became larger because the attributable deaths from these risk factors occurred among young adults aged less than 60 years.

**Conclusions:**

High blood glucose, tobacco smoking, and high blood pressure are the major risk factors for deaths from diseases and injuries among Taiwanese adults. A large number of years of life would be gained if the 13 modifiable risk factors could be removed or reduced to the optimal level.

**Electronic supplementary material:**

The online version of this article (doi:10.1186/s12963-017-0134-4) contains supplementary material, which is available to authorized users.

## Background

Quantitative analyses on how different risk factors contribute to the overall disease burden provide critical information for health policymaking and priority-setting. The comparative risk assessment approach developed under the Global Burden of Diseases, Injuries, and Risk Factors Study (GBD) provides a framework for population risk assessment and comparison across risks at the global and national levels [1, 2]. However, the country-level analyses from GBD may suffer from the problem of data gaps when information on disease outcomes or risk factors is not available [3]. Meanwhile, despite the inclusion of a large number of disease outcomes and risk factors in the GBD study, there are still locally important diseases and risk factors which are not included in GBD (e.g., betel nut use in South and Southeast Asia). Therefore the country-level results from the GBD analysis may not represent the best available evidence for the purpose of local health policymaking.

Independent national analyses based on the specific local public health context will complement the current GBD analysis. In Taiwan, previous studies have reported the attributable disease burden due to single modifiable risk factors, but none has included all major risk factors using a comprehensive and comparable approach [4–9]. In addition, Taiwan is not always included as a separate entity in the GBD analysis. Taking advantage of the well-established health information system in Taiwan, we estimated the mortality burden attributable to 13 metabolic, lifestyle, infectious, and environmental risk factors.

## Methods

**Fig. 1:**
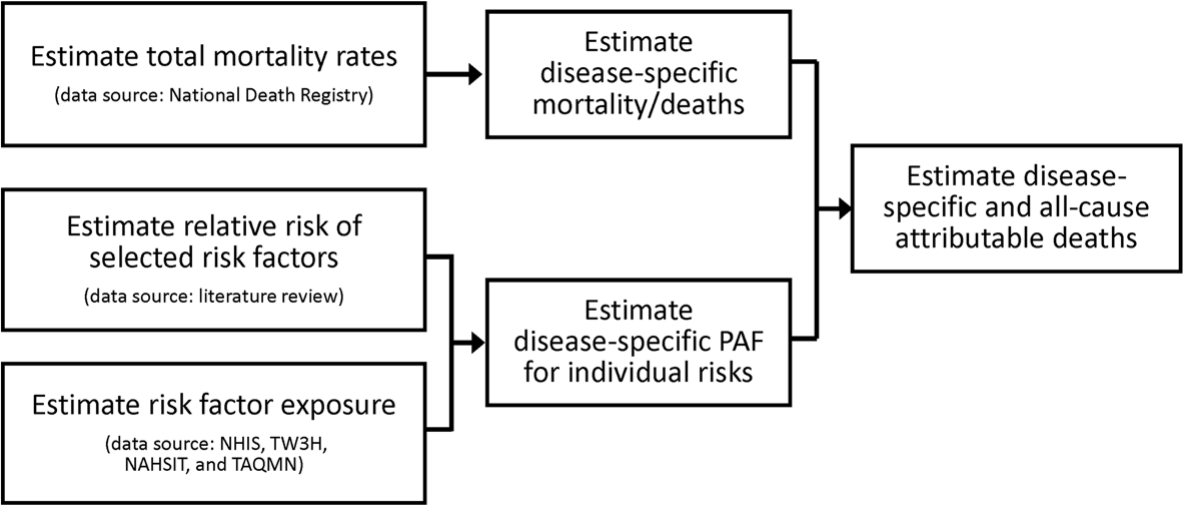
Principle component steps of comparative risk assessment. NHIS: National Health Interview Survey; TW3H: Taiwanese Survey on Blood Sugar, Blood Lipids and Blood Pressure; NAHSIT: Nutrition and Health Survey in Taiwan; TAQMN: Taiwan Air Quality Monitoring Network; PAF: population-attributable fraction

We employed the comparative risk assessment framework to estimate the number of deaths and years of life lost (YLLs) for adults aged over 30 years attributable to major risk factors in Taiwan in 2009 [10, 11]. The general framework and data sources are presented in Fig. 1. We first computed the population-attributable fraction (PAF) of cause-specific mortality for each risk factor. For risk factors measured in multiple categories, we used the following generalized formula to calculate PAFs:

$$ P A F=\frac{{\displaystyle {\sum}_{i=1}^n}{P}_i\left( R{R}_i-1\right)}{{\displaystyle {\sum}_{i=1}^n}{P}_i\left( R{R}_i-1\right)+1} $$

where *i* represents the level of exposure categories (i = 1,…, n); *RR*_*i*_ is the relative risk for exposure category *i*; *P*_*i*_ is the proportion of the population in exposure category *i* [12]. For risk factors measured continuously (e.g., blood pressure and blood glucose), the following generalized formula was used to compute PAFs:

$$ P A F=\frac{{\displaystyle {\int}_x RR(x) P(x) dx}-{\displaystyle {\int}_x RR(x) P\hbox{'}(x) dx}}{{\displaystyle {\int}_x RR(x) P(x) dx}} $$

where *RR(x)* is the relative risk at exposure level *x*; *P(x)* is the actual distribution of risk factor exposure in the population; *P’(x)* is the counterfactual distributions of risk factor exposure. Using the PAF approach, we estimated the population-level effects of all risk factors in a consistent and comparable way [13]. We summed the number of deaths and the number of YLLs attributable to a single risk factor across different causes to obtain the total number of deaths and YLLs attributable to the risk factor of interest.

### Selection of diseases and risk factors

Leading causes of death in Taiwan were selected as the disease outcome in our analysis (Additional file 1: Table S1). We included 13 major modifiable risk factors in the analysis according to predefined criteria, including four metabolic risk factors (high blood pressure, high total cholesterol, high blood glucose, and overweight-obesity [high body mass index, BMI]), four lifestyle risk factors (physical inactivity, tobacco smoking, alcohol use, and betel nut use), two dietary risk factors (high sodium intake and low intake of fruits and vegetables), one environmental risk factor (ambient particulate matter pollution), and two infectious risk factors (hepatitis B virus [HBV] infection, and hepatitis C virus [HCV] infection). (See Additional file 1 for details). In the present study, we selected infections from HBV and HCV and betel nut use as domestic risk factors. These risk factors were not included in the GBD comparative risk assessment but were highly prevalent and may cause substantial burden of disease in Taiwan.

### Mortality data

The numbers of cause-specific deaths were obtained from the National Death Registry. The major disease outcomes were defined by ICD-10 code (Additional file 1: Table S1). We used the Multiple Cause of Death Data to redistribute the “garbage codes” which are the causes of death that cannot or should not be considered the underlying cause of death (see Additional file 1 for details) [14]. We calculated YLLs by summing the number of fatal cases multiplied by the residual expected life expectancy based on the standard life tables from GBD 2013 [2].

### Exposure to risk factors

Risk factor exposure distributions were obtained from nationally representative health surveys. We used the 2009 National Health Interview Survey (NHIS) to quantify the exposure to passive tobacco smoking, alcohol use, betel nut use, physical inactivity, and overweight-obesity. Data from the 2007 Taiwanese Survey on Blood Sugar, Blood Lipids, and Blood Pressure (TW3H) were used to evaluate the distribution of blood pressure, total cholesterol, blood glucose, and HBV and HCV infection. We used the smoking impact ratio (SIR) to measure the cumulative exposure to tobacco smoking [15, 16] (see Additional file 1 for details).

We used the 2013 Nutrition and Health Survey in Taiwan (NAHSIT) to determine the distribution of dietary risks (high sodium intake and low intake of fruits and vegetables). We obtained data on particles measuring less than 2.5 μm in diameter (PM_2.5_) from Taiwan Air Quality Monitoring Network established by the Taiwan Environmental Protection Administration (EPA) [17].

We used an optimal distribution which has minimal harmful effects on morbidity and mortality (theoretical minimum-risk exposure distribution, TMRED) as an alternative distribution of risk exposures to measure the mortality effects of actual exposure of risk factors (Table 1) (see Additional file 1 for details) [18].

**Table 1:** Measurements, data sources, theoretical minimum-risk exposure distributions (TMRED), and corresponding disease outcome. The numbers for alternative exposure distribution represent mean and standard deviation

Risk factor	Exposure Metric	TMRED	Data Source	Disease Outcome
High blood pressure	Systolic blood pressure (mmHg)	115 (6)	TW3H 2007	IHD, stroke, hypertensive heart diseases, other cardiovascular diseases^a^
High total cholesterol	Total cholesterol (mg/dL)	147 (23.2)	TW3H 2007	IHD, ischemic stroke
High blood glucose	Fasting plasma glucose (mg/dL)	88.2 (5.4)	TW3H 2007	IHD, stroke, diabetes mellitus, CKD
Overweight/obesity	Body mass index (kg/m^2^)	21 (1)	NHIS 2009	IHD; ischemic stroke; hypertensive heart disease; other cardiovascular diseases^a^; breast, colon, and kidney cancers; diabetes mellitus
Sodium intake	Daily intake (g/d)	0.5 (0.05)	NAHSIT 2013	IHD, stroke, hypertensive heart diseases, other cardiovascular diseases^a^
Fruit and vegetable intake	Daily intake (g/d)	600 (50)	NAHSIT 2013	IHD; ischemic stroke; colorectal, stomach, lung, oral, esophageal cancers
Tobacco smoking	Smoking Impact Ratio	No smoking	NHIS 2001, NHIS 2005, and Civil Servant cohorts	IHD; stroke; other cardiovascular diseases; cancers of lung and selected other sites (see Additional file 1 for details); CKD; COPD
Alcohol use	Current alcohol use status	No alcohol use	NHIS 2009	IHD; ischemic stroke; hemorrhagic stroke; hypertensive heart diseases; cancers of mouth, esophagus, breast, liver, and selected other sites (see Additional file 1 for details); diabetes mellitus; chronic liver disease; alcohol abuse; road traffic injuries; suicide
Betel nut use	Current betel nut use status	No betel nut use	NHIS 2009	Oral, esophagus, and larynx cancers
Physical inactivity	Intensity of physical activity	Have intense physical activity	NHIS 2009	IHD, ischemic stroke, breast and colon cancers, diabetes mellitus
Ambient particulate matter pollution	PM 2.5 (μg/m^3^)	7.5 (0.75)	TAQMN 2009	IHD, stroke, lung cancer, COPD
Hepatitis B virus	Seropositivity for hepatitis B surface antigen	No infection	TW3H 2007	Liver cancer, chronic liver disease
Hepatitis C virus	Seropositivity for antibody to hepatitis C	No infection	TW3H 2007	Liver cancer, chronic liver disease

*TW3H* Taiwanese survey on blood sugar, blood lipids and blood pressure, *NHIS* National Health Interview Survey, *NAHSIT* Nutrition and Health Survey in Taiwan

*TAQMN* Taiwan Air Quality Monitoring Network, *IHD* ischemic heart disease, *CKD* chronic kidney disease, *COPD* chronic obstructive pulmonary disease

^a^: Other cardiovascular diseases (ICD 10: I00, I26–I28, I34–I37, I44–I51, I70–I99)

### Selection of relative risks and joint effects of multiple risk factors

The relative risks (RRs) for each exposure-disease pair were obtained from systematic reviews in previous comparative risk assessment studies or best available evidence for the purpose of the study. Since the cardiometabolic risk factors of high blood pressure, high blood glucose, high total cholesterol, and high BMI are often correlated with each other at the population level and the effects of BMI on cardiovascular diseases (CVDs) have been shown to be mediated by other risk factors [19, 20], we estimated the joint effect of these cardiometabolic risk factors on CVDs, accounting for risk factor correlation and mediation effects (see Additional file 1 for details).

### Uncertainty analyses

To deal with the uncertainty due to sampling variability, we took a statistical simulation approach [21]. We randomly drew 1,000 sets of risk exposures and RRs from their distributions. Each set of sampled risk exposure and RR was used to compute the PAF and the number of deaths attributable to each risk factor, separately by age and sex. We reported 95% uncertainty intervals (UIs) by a span across the estimates of each outcome at the 2.5th and 97.5th percentiles based on the resulting distributions of 1,000 estimated attributable deaths. Analyses were performed using the statistical software Stata, version 10.1. The statistical source code used to generate estimates can be accessed upon request to the authors.

## Results

According to the National Death Registry, there were 143,582 deaths in Taiwan in 2009. Of these, 61% were men, and 138,984 deaths occurred in adults aged 30 years or over. The total number of deaths with garbage codes was 22,659, accounting for 15.8% of total deaths in 2009. After redistribution of garbage codes, the leading causes of death were diabetes (10,160 deaths), ischemic heart disease (IHD) (9,380 deaths), and lung cancer (8,940 deaths). A total of 110,720 deaths from 12 causes were included in this study.

**Fig. 2:**
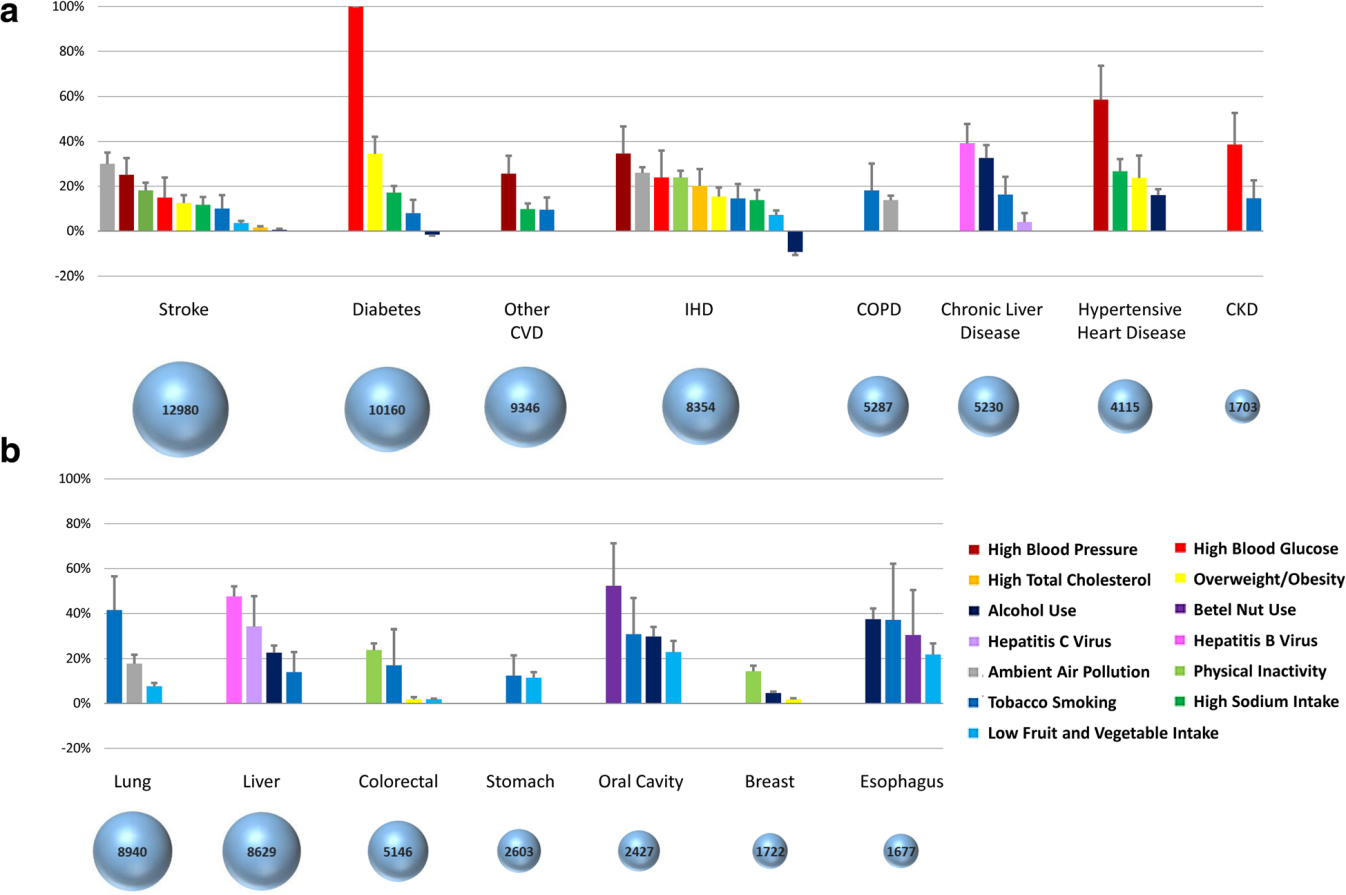
The ranking of PAFs of selected risk factors for cardiovascular diseases and other non-communicable diseases (**a**); and cancers by site (**b**). The bubble size represents the total number of deaths from each cause. IHD: ischemic heart disease; CVD: cardiovascular disease; CKD: chronic kidney disease; COPD: chronic obstructive pulmonary disease

The distributions of risk factors are presented in the Additional file 1: Tables S2–S4. Using the risk factor exposures and the exposure-disease associations from the literature, we estimated the PAF due to different risk factors for each cause of death (Fig. 2). For diseases other than cancer, ambient air pollution, high blood glucose, and high blood pressure were the risk factors with the largest cause-specific PAFs. For cancer by sites, the leading risk factors were tobacco smoking, viral hepatitis, physical inactivity, and betel nut use.

**Fig. 3:**
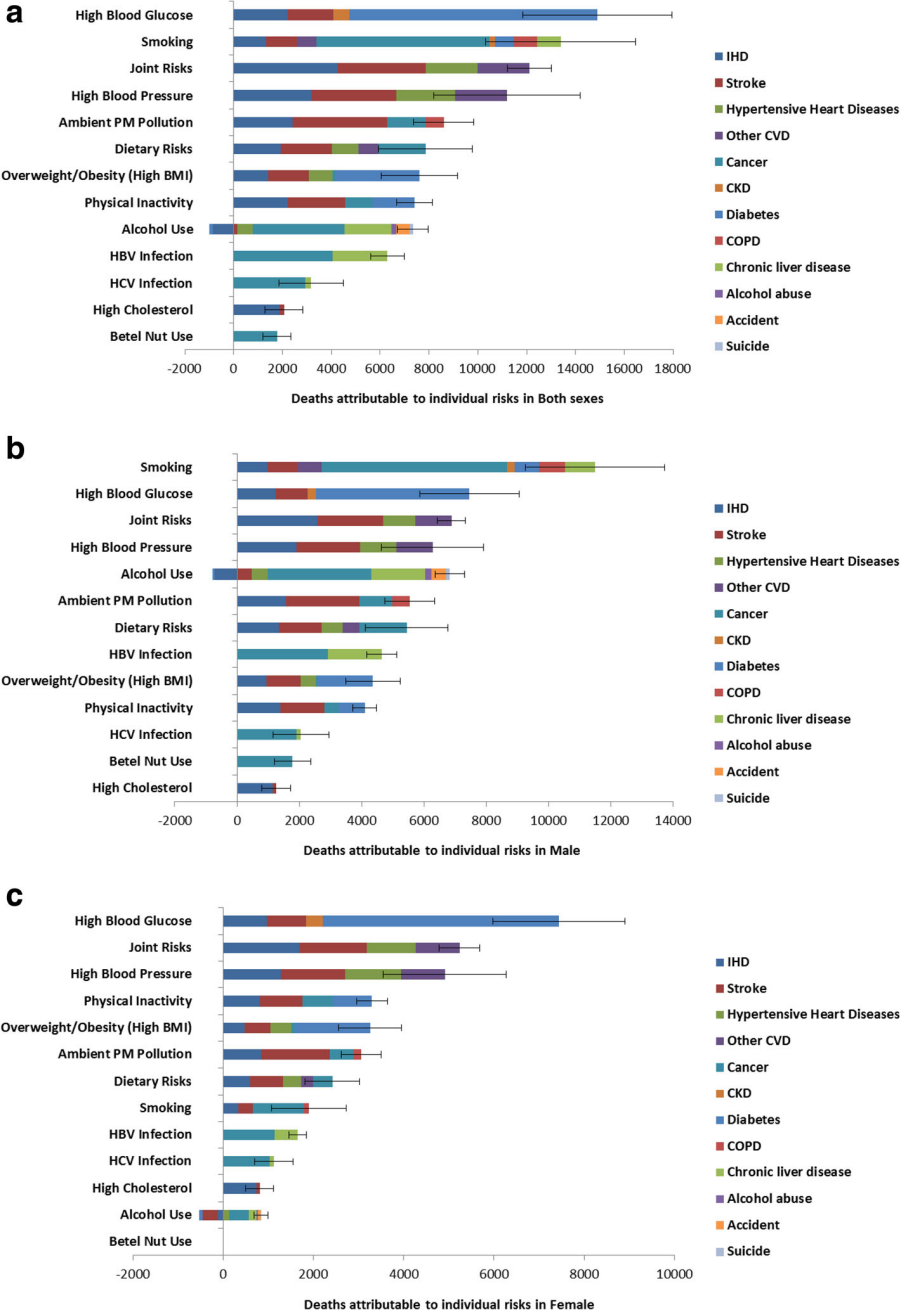
Deaths attributable to modifiable risk factors by cause in both sexes (**a**), in men (**b**), and in women (**c**) IHD: ischemic heart disease; CVD: cardiovascular disease; CKD: chronic kidney disease; COPD: chronic obstructive pulmonary disease; BMI: body mass index

Among the 13 risk factors, high blood glucose accounted for the largest number of deaths (14,900, 95% UI: 11,850–17,960) in Taiwan in 2009, equivalent to 64.6 deaths per 100,000 population or 10.4% of total deaths in the year (Table 2, Fig. 3, and Additional file 1: Figure S1). The cause of death was diabetes in 68.2% of these deaths. The mortality burden from high blood glucose was distributed evenly in both sexes. More than 80% of deaths due to high blood glucose occurred in people older than 65 years.

**Table 2:** The number of deaths attributable to selected risk factors in Taiwan, 2009

Risk factor	Total	(95% UI)	CVD^a^	(95% UI)	Cancer	(95% UI)	Diabetes	(95% UI)	Chronic liver disease	(95% UI)	Other NCDs and injury^b^	(95% UI)
Overall												
High blood pressure	11,190	(8,190–14,190)	11,190	(8,190–14,190)								
High total cholesterol	2,070	(1,300–2,830)	2,070	(1,300–2,830)								
High blood glucose	14,900	(11,850–17,960)	4,090	(2,120–6,070)			10,160				650	(410–890)
Overweight/obesity	7,610	(6,040–9,190)	4,050	(3,060–5,050)	120	(60–190)	3,440	(2,700–4,170)				
Dietary risks	7,890	(5,970–9,810)	5,950	(4,500–7,410)	1,940	(1,470–2,410)						
Tobacco smoking	13,400	(10,330–16,450)	3,390	(1,590–5,170)	7,060	(3,420–10,680)	790	(230–1,340)	970	(490–1,430)	1,190	(420–1,930)
Alcohol use	6,350	(5,730–6,970)	−60^c^	(−250–130)	3,770	(3,550–4,000)	−150^c^	(−190– − 110)	1,910	(1,680–2,130)	880	(800–960)
Betel nut use	1,780	(1,190–2,360)			1,780	(1,190–2,360)						
Physical inactivity	7,400	(6,670–8,130)	4,560	(3,990–5,120)	1,130	(950–1,300)	1,710	(1,370–2,060)				
Ambient PM pollution	8,600	(7,370–9,840)	6,290	(5,470–7,110)	1,580	(1,290–1,870)					730	(610–850)
Hepatitis B virus	6,300	(5,610–6,980)			4,050	(3,680–4,410)			2,250	(1,700–2,800)		
Hepatitis C virus	3,170	(1,860–4,490)			2,940	(1,730–4,150)			230	(20–450)		
Joint cardiometabolic risks	12,120	(11,220–13,020)	12,120	(11,220–13,020)								
Men												
High blood pressure	6,280	(4,640–7,920)	6,280	(4,640–7,920)								
High total cholesterol	1,260	(800–1,720)	1,260	(800–1,720)								
High blood glucose	7,460	(5,870–9,050)	2,260	(1,160–3,360)			4,920				280	(170–380)
Overweight/obesity	4,360	(3,480–5,240)	2,540	(1,950–3,130)	50	(20–80)	1,770	(1,400–2,140)				
Dietary risks	5,460	(4,150–6,770)	3,950	(3,000–4,910)	1,510	(1,140–1,870)						
Tobacco smoking	11,500	(9,260–13,730)	2,720	(1,330–4,110)	5,950	(3,200–8,700)	790	(230–1,340)	960	(490–1,430)	1,080	(470–1,690)
Alcohol use	6,030	(5,560–6,500)	260	(160–370)	3,330	(3,160–3,500)	−70^c^	(−100– −40)	1,730	(1,560–1,900)	780	(710–840)
Betel nut use	1,780	(1,190–2,360)			1,780	(1,190–2,360)						
Physical inactivity	4,100	(3,720–4,490)	2,800	(2,470–3,120)	450	(390–510)	850	(690–1,020)				
Ambient PM pollution	5,550	(3,530–5,740)	3,920	(3,420–4,430)	1,060	(860–1,250)					570	(470–660)
Hepatitis B virus	4,650	(4,150–5,130)			2,920	(2,670–3,160)			1,730	(1,330–2,130)		
Hepatitis C virus	2,050	(1,150–2,950)			1,910	(1,080–2,750)			140	(10–270)		
Joint cardiometabolic risks	6,880	(6,430–7,330)	6,880	(6,430–7,330)								
Women												
High blood pressure	4,910	(3,550–6,270)	4,910	(3,550–6,270)								
High total cholesterol	810	(500–1,120)	810	(500–1,120)								
High blood glucose	7,440	(5,980–8,910)	1,830	(960–2,710)			5,240				370	(240–510)
Overweight/obesity	3,250	(2,560–3,950)	1,520	(1,120–1,920)	70	(40–110)	1,660	(1,300–2,030)				
Dietary risks	2,430	(1,820–3,040)	2,000	(1,490–2,500)	430	(330–540)						
Tobacco smoking	1,900	(1,070–2,720)	660	(260–1,060)	1,140	(220–1,980)					100	(−40–250)
Alcohol use	320	(160–470)	−320^c^	(−410– − 230)	440	(390–490)	−80^c^	(−90– − 70)	180	(120–230)	100	(90–110)
Betel nut use	0											
Physical inactivity	3,300	(2,950–3,640)	1,760	(1,520–2,000)	680	(560–780)	860	(680–1,040)				
Ambient PM pollution	3,050	(2,610–3,500)	2,370	(2,050–2,680)	520	(420–620)					160	(140–190)
Hepatitis B virus	1,650	(1,460–1,850)			1,130	(1,020–1,250)			520	(370–670)		
Hepatitis C virus	1,120	(700–1,540)			1,030	(650–1,400)			90	(10–180)		
Joint cardiometabolic risks	5,240	(4,790–5,680)	5,240	(4,790–5,680)								

^a^ *CVD* cardiovascular diseases, ^b^ *NCD* non-communicable disease, other NCDs include chronic obstructive pulmonary disease, chronic kidney disease, alcohol abuse; injury: accidental injury and suicide

^c^Moderate alcohol consumption has been shown to reduce the risk of death from some causes, resulting a negative numbers of attributable deaths in our analysis

Tobacco smoking was responsible for 11,500 deaths in men and 1,900 deaths in women, or 9.3% of all deaths. Active smoking contributed to 83% of smoking-attributable deaths. In men, 60% of smoking-associated deaths were caused by cancers and 95.9% of smoking-associated deaths occurred in those over 45 years old. In women, the attributable mortality from smoking was low (4.6%) because of the low prevalence of smoking in women. For both sexes combined, lung cancer was the leading cause among smoking-attributable deaths (3,660 deaths, 95% UI: 2,340–4,980), followed by IHD (1,320 deaths, 95% UI: 760–1,880) and stroke (1,280 deaths, 95% UI: 540–2,020).

High blood pressure was associated with 11,190 deaths from CVDs (95% UI: 8,190–14,190), or 7.8% of all deaths in year 2009. The majority of deaths attributable to high blood pressure occurred among people aged 65 years and over (8,500 deaths). Among the deaths attributable to high blood pressure, the leading causes were stroke (3,470 deaths, 95% UI: 2,420–4,520), IHD (3,190 deaths, 95% UI: 2,020–4,350), and hypertensive heart disease (2,400 deaths, 95% UI: 1,760–3,050).

The cluster of cardiometabolic risk factors, including high blood pressure, high blood glucose, high cholesterol, and overweight-obesity, accounted for 12,120 cardiovascular deaths (95% UI: 11,220–13,020) in 2009. Other leading risk factors included ambient particulate matter pollution (PM_2.5_) (8,600 deaths, 95% UI: 7,370–9,840), high sodium intake and low intake of fruits and vegetables (7,890 deaths, 95%UI: 5,970–9,810), physical inactivity (7,620 deaths, 95% UI: 6,040–9,190), and overweight-obesity (7,400 deaths, 95% UI: 6,670–8,130). Alcohol use accounted for 6,350 deaths (95% UI: 5,730–6,970) from non-communicable diseases (cancer and chronic liver disease) and injury (accident and suicide). HBV infection and HCV infection accounted for 6,300 (95% UI: 5,610–6,980) and 3,170 (95% UI: 1,860–4,490) deaths, respectively. Under the assumptions of no correlation and no effect mediation, HBV and HCV infection and alcohol use jointly accounted for 72.4% (6,250) of deaths from liver cancer and 58.6% (3,420) of deaths from chronic liver disease. Lastly, high cholesterol was associated with 2,070 CVDs deaths (95% UI: 1,300–2,830), and betel nut use was associated with 1,780 deaths (95% UI: 1,190–2,360) from oral, laryngeal, and esophageal cancer.

**Fig. 4:**
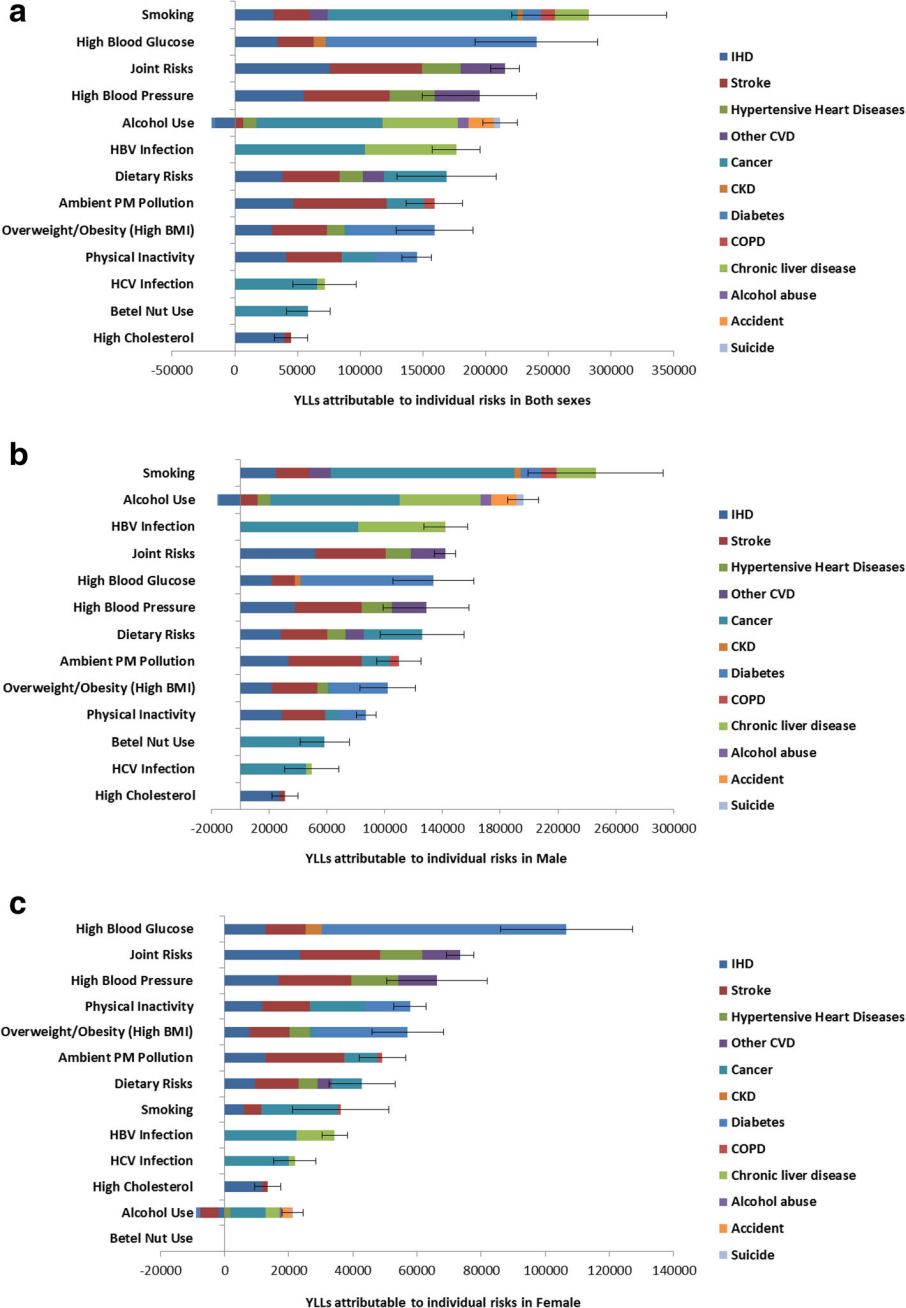
Years of life lost (YLLs) attributable to modifiable risk factors by cause in both sexes (**a**), in men (**b**), and in women (**c**) IHD: ischemic heart disease; CVD: cardiovascular disease; CKD: chronic kidney disease; COPD: chronic obstructive pulmonary disease; BMI: body mass index

The analysis of attributable YLLs revealed a slightly different pattern for risk factor ranking (Fig. 4 and Additional file 1: Figure S2). Tobacco smoking became the leading cause of attributable YLLs (246,030 YLLs for men and 36,310 YLLs for women), accounting for 1,223 YLLs per 100,000 population in 2009. This was followed by high blood glucose (240,450 YLLs, 95%UI: 191,820–289,090) and joint cardiometabolic risk factors (215,540 YLLs, 95%UI: 204,020–227,050).

## Discussion

Using information from nationally representative surveys and vital registry, we estimated and compared the impacts of modifiable risk factors on major causes of mortality in Taiwan. We found a high mortality burden attributable to high blood glucose, tobacco smoking, and high blood pressure. Notably, our analysis revealed the importance of several domestic risk factors that were not included in the GBD study’s comparative risk assessment: HBV and HCV infection, and betel nut use.

The ranking of risk factors in our analysis was similar to that from the Taiwan analysis of GBD 2015, but we note that the comparison of two analyses should be made carefully [1]. In GBD 2015, systematic reviews and statistical modeling were used to generate age- and sex-specific exposure distributions for each risk factor in each country, while our study exclusively used data from nationally representative surveys. In addition, the redistribution of garbage codes in our analysis was based on Multiple Cause of Death Data instead of the single cause of death method used in GBD 2015. As a result, we found that the absolute numbers of deaths attributable to risk factors in our analysis were very different from the 2010 estimates of GBD 2015 (Additional file 1: Table S5). For example, the attributable number of deaths from high blood pressure, tobacco smoking, and alcohol use were two times higher in the 2010 estimates from GBD 2015 than those in our analysis.

HBV and HCV infection and betel nut use did not appear in the comparative risk assessments of GBD studies (viral hepatitis was included directly as a disease cause instead of a risk factor in GBD) [1, 2, 22]. We included these risk factors in the present analysis because they are highly prevalent in the Taiwanese population and are preventable (through HBV vaccination) and modifiable (through lifestyle change or antiviral treatment) [23]. Liver diseases have long been considered the “disease of the country” for the Taiwanese population [24]. Our analysis revealed that HBV and HCV infection accounted for a large number of deaths from liver cancer and chronic liver disease. We also found that the population mortality impact of betel nut use was at least as large as that of high cholesterol. Our results suggested that viral hepatitis and betel nut use should be considered in the comparative risk assessment in countries where these risk factors are prevalent. For example, the prevalence of betel nut use is up to 40% in certain Southeast Asian countries (men in Nepal and women in Indonesia), and HBV affects 5.26% of the population in the WHO Western Pacific Region [25, 26].

In our analysis, high blood glucose accounted for the largest mortality burden among all risk factors. The ranking of high blood glucose in Taiwan was particularly high compared with other countries [1, 2]. We note that the finding should be interpreted with caution since 68% of this mortality burden came from diabetes as the cause of death. Previous studies revealed substantial differences in diabetes-related cause of death statements among physicians in Taiwan compared with other countries [27]. Therefore, in addition to the true differences in deaths attributable to high blood glucose, the difference in ranking may also reflect the country-specific practice in recording cause of death. Nonetheless, the high disease burden from high blood glucose and the rising trend of diabetes prevalence call for urgent actions to curb this epidemic [28].

We found that tobacco smoking was the leading risk factor for YLLs in Taiwan and the leading risk factor for mortality in Taiwanese men. This is consistent with previous studies that reported a large mortality burden from smoking in Taiwan [4, 29]. Despite the continued reduction in smoking prevalence in the past decade, the mortality burden from smoking remained high in our analysis [30]. It should also be noted that the recent reduction in smoking prevalence was mainly observed in the elderly male population [31]. Future trends of smoking prevalence in all age and sex groups need to be closely monitored.

High blood glucose, high blood pressure, high BMI, and high cholesterol jointly accounted for 12,120 deaths from CVD, or 8.4% of all deaths. To reduce the disease burden from these risk factors, the Taiwan Health Promotion Administration launched a series of programs, including enhancing preventive health care services for the elderly, building a health-supportive environment, promoting a daily walking program, and initiating healthy exercise for workers [32]. Further evaluations would be critical to determine the cost-effectiveness of these programs.

Our subnational analysis of ambient PM_2.5_ pollution revealed that nearly 9,000 deaths could be attributed to ambient particulate matter pollution every year, and the causes of death were mainly CVD, lung cancer, and COPD. Although PM_2.5_ exposure has been decreasing in recent years, the current level of PM_2.5_ exposure is still far from the optimal level that has the minimal health risk [33, 34]. A coordinated, multisectoral effort that involves at least the Ministry of Health and Welfare, Environmental Protection Agency (EPA), and Ministry of Economic Affairs will be needed to address the health effects from ambient air pollution.

As with other PAF studies, we urge caution in the interpretation and policy implications of the estimates from the present analysis. First, by definition the PAF represents the proportional reduction of disease burden if the exposure of interest were eliminated from the population, *while nothing else changed*. The implicit assumption of the PAF approach is that the risk factor of interest can be modified without changing the distributions of all other risk factors. This assumption is not well-justified for most cardiometabolic risk factors. For example, it is difficult to imagine any intervention that would eliminate high blood glucose without changing the prevalence of obesity and the distribution of blood pressure in the population. We therefore estimated the joint attributable burden of CVD deaths from cardiometabolic risk factors. Second, the magnitude of attributable deaths does not imply the level of resources required for one particular risk factor, nor does the ranking of risk factors directly determine prioritization in health policymaking. Further cost-effectiveness analysis and health technology assessment should be conducted on available interventions for these risk factors while taking into account the affordability of each intervention.

Our study has limitations. First, as with other comparative risk assessment studies, our analysis used the cross-sectional information on risk factor exposure and mortality outcomes in the same year. This would introduce bias if the risk factor exposure has changed substantially over time. We used the SIR to account for the cumulative exposure of tobacco smoking, but not for other risk factors. Second, occupational risk factors and some dietary risks were not included. Third, our study focused on attributable mortality burden without consideration of morbidity and disability.

Our integrative analysis provides an aerial view of distribution and determinants of population health and helps the country to prepare for the new Sustainable Development Goals (SDGs) [35]. Our findings suggest that achieving the health-related SDG (SDG 3) requires collaborative efforts from the communicable and non-communicable health sectors as well as non-health sectors (e.g., the EPA and the Ministry of Economic Affairs for clean energy, SDG 7), highlighting the importance of partnerships for sustainable development (SDG 17). Our analysis echoes the SDGs and indicates that there are multiple entry points to improving population health and tremendous needs for an integrated approach. In the meantime, operational and technological innovations are sorely needed to help countries to move from the current siloed approach to an integrated approach.

## Conclusions

Our analysis of Taiwan revealed that substantial mortality burden and premature deaths could be attributable to cardiometabolic risk factors, tobacco smoking, alcohol use, viral hepatitis, and ambient PM pollution. Aggressive interventions to reduce these risk factors have the potential to save tens of thousands of years of life lost. Compared with the findings from the GBD analysis, our analysis revealed quantitative differences in mortality attributable to risk factors. This highlights the importance of data-driven national analyses in the GBD era with the aim to inform evidence-based local health policymaking.

## Additional file

Additional file 1: Detailed information about the methods used in this study and results of additional analyses. (DOCX 445 kb)

## Abbreviations

BMI: Body mass index
CVD: Cardiovascular disease
EPA: Taiwan environmental protection administration
GBD: Global burden of diseases, injuries, and risk factors study
HBV: Hepatitis B virus
HCV: Hepatitis C virus
IHD: Ischemic heart disease
NAHSIT: Nutrition and health survey in Taiwan
NHIS: National health interview survey
PAF: Population-attributable fraction
PM_2.5_: Particles measuring less than 2.5 μm in diameter
RRs: Relative risks
SDGs: Sustainable development goals
SIR: Smoking impact ratio
TMRED: Theoretical minimum-risk exposure distribution
TW3H: Taiwanese survey on blood sugar, blood lipids and blood pressure
UIs: Uncertainty intervals
YLLs: Years of life lost
