# Supplementary material for: Adult mortality of diseases and injuries attributable to selected metabolic, lifestyle, environmental, and infectious risk factors in Taiwan: a comparative risk assessment
Source: Popul Health Metr. 2017 May 3;15:17. doi: 10.1186/s12963-017-0134-4 (PMC5415794; doi:10.1186/s12963-017-0134-4)
Supplement: Supplementary file 1 — Detailed information about the methods used in this study and results of additional analyses. (DOCX 445 kb) [file 12963_2017_134_MOESM1_ESM.docx]

**Appendix**

This appendix provides detailed information about the methods used in this study and results of additional analyses.

**Selection of risk factors and estimation of risk exposure**

Thirteen risk factors were selected according to the following criteria: (i) the likelihood of causality or association is high based on well-designed and high-quality epidemiological studies; (ii) the risk exposures are potentially modifiable with interventions; (iii) the risk factors are responsible for the leading causes of disease burden at the national level; (iv) valid and complete data from nationally representative surveys on risk exposure are available [[1](#_ENREF_1)]{Report, 2002 #13}{Report, 2002 #13}{Report, 2002 #13;Report, 2002 #13}.

Risk factor exposure distributions were obtained from three nationally representative health surveys. Body mass index (BMI) and lifestyle risk factors (physical inactivity, alcohol use, and betel nut use) were collected from the National Health Interview Survey (NHIS). Taiwanese Survey on Blood Sugar, Blood Lipids and Blood Pressure (TW3H) provided information on metabolic risk factors (high blood pressure, high total cholesterol, and high blood glucose) and infectious risk factors including hepatitis B virus (HBV) and hepatitis C virus (HCV) infection. Nutrition and Health Survey in Taiwan (NAHSIT) were used to estimate the distribution of dietary risks (high sodium intake and low intake of fruits and vegetables).

In the NHIS, physical activity was recorded by activity type, frequency, and duration. In order to standardize the assignment of physical activity status in epidemiology studies, a simple metabolic equivalent (MET) calculation has been developed and used as a [physiological](http://en.wikipedia.org/wiki/Physiological) measure expressing the energy cost of [physical activities](http://en.wikipedia.org/wiki/Exercise). According to the compendium of physical activities project, we assigned every [physical activity](http://en.wikipedia.org/wiki/Exercise) with appropriate METs, and classified it into moderate-intensity or vigorous-intensity activity [[2](#_ENREF_2)]. The definition of moderate-intensity activity was METs=3-6, and vigorous-intensity activity was METs>6 [[3](#_ENREF_3)]. We adopted the category defined by Bull FC et.al [[4](#_ENREF_4)] to assign physical inactivity exposure into three levels: inactive (no moderate or vigorous activity), insufficiently active (<2.5h/wk of moderate activity or < 1h/wk of vigorous activity), sufficiently active (≥2.5 hr/wk of moderate activity or ≥1 hr/wk of vigorous activity).

The indirect indicator of smoking impact ratio (SIR) was used to measure cumulative exposure to tobacco smoking [[5](#_ENREF_5), [6](#_ENREF_6)]. The SIR quantifies the excess lung cancer mortality in the study population relative to the excess lung cancer mortality in current smokers. We combine the NHIS 2001, the NHIS 2005, and the civil servant cohorts with National Death Registry to estimate SIR.

$$Smoking impact ratio=\frac{C_{LC}-N_{LC}}{{S^{*}}_{LC}-{N^{*}}_{LC}} \times\frac{{N^{*}}_{LC}}{N_{LC}}$$

where *C_LC_* and *N_LC_* represent lung cancer mortality in the total population and the never smokers, respectively, in the study population, and *S*^*^*_LC_* and *N*^*^*_LC_* represent lung cancer mortality of current and never smokers in the reference population from which the relative risks were derived. The *C_LC_* estimates were obtained from National Death Registry dataset, while *N_LC_* estimates were obtained from NHIS 2001, NHIS 2005, and civil servant cohorts with 10-year follow-up. We also estimated lung cancer mortality by smoking status in the reference population (NHIS 2001, NHIS 2005, and civil servant cohorts) [[7-10](#_ENREF_7)]. The SIR estimates for men aged over 80 years and women in NHIS cohort became unstable due to the low prevalence of tobacco smoking and few events. We therefore used the prevalence of tobacco smoking estimated from NHIS among these age groups.

Data on drinking patterns were collected in quantitative format with types of liquor and drinking frequency in the NHIS 2009. We transformed these data into average volume of alcohol consumption per day. According to the GBD report and previous meta-analyses, we classified alcohol consumption into four levels: 1) abstainer: a person not having had a drink containing alcohol within the last year; 2) drinking category I: for females 0–19.99 g pure alcohol daily; for males 0–39.99 g pure alcohol daily; 3) drinking category II: for females 20–39.99 g pure alcohol daily; for males 40–59.99 g pure alcohol daily; and 4) drinking category III: for females 40 g or more pure alcohol daily; for males 60 g or more pure alcohol daily [[11](#_ENREF_11), [12](#_ENREF_12)].

As a nationwide population-based survey, the TW3H collected profiles of high blood pressure, hyperglycemia, and high blood lipid among 6,600 subjects in Taiwan. Study subjects of TW3H were randomly selected from the NHIS 2001, which incorporated a multi-staged stratified systematic sampling scheme. Measurement of blood pressure, fasting blood lipids, and fasting glucose followed standardized procedures. Also, the blood samples were used to determine the prevalence of HBsAg and anti-HCV antibody.

On the other hand, government-sponsored NAHSIT 2013-2014 adopted a multistaged, stratified, and clustered probability design to provide a nationally representative estimation in nutrition and related health conditions. Dietary sodium, fruit, and vegetable intake was estimated from 24-hour dietary recalls. Data were gathered from 2,911 subjects aged 19 years and older who had completed the dietary assessment.

For ambient air pollution, measurement of PM_2.5_ was obtained from Taiwan Air Quality Monitoring Network, which was established by the Taiwan Environmental Protection Administration (EPA) in 1993. A total of 76 air quality stations and four mobile sites were set up to collect hourly measurements of CO, NOx, SO_2_, PM, and O_3_ to provide nationally representative air pollution data [[13](#_ENREF_13)].

**Counterfactual Distributions of Risk Exposures**

For metabolic risk factors (BMI, blood pressure, blood glucose, and total cholesterol), zero exposure is physiologically impossible. We set theoretical minimum-risk exposure distribution, TMRED, based on the levels corresponding to the lowest mortality rate in epidemiological studies for these risk factors [[14](#_ENREF_14)]. Furthermore, we assigned the TMREDs of tobacco smoking, betel nut use, alcohol use, air pollution, and infections to zero exposure, even though moderate consumption of alcohol might be beneficial for cardiovascular disease [[11](#_ENREF_11), [15](#_ENREF_15)].

**Selection of Relative Risks**

All relative risks applied to our analyses were quantified associations with mortality. For metabolic risk factors (high blood pressure, high total cholesterol, high blood glucose, high BMI), physical inactivity, dietary risks (high sodium intake and low intake of fruits and vegetables), passive smoking, and alcohol use, we used the evidence synthesized from previous comparative risk assessment (CRA) studies and meta-analyses [[5](#_ENREF_5), [12](#_ENREF_12), [16-22](#_ENREF_16)]. For ambient particulate matter pollution (PM_2.5_), the integrated exposure-response relative risk (RR) functions between ambient PM_2.5_ and specific causes of deaths were based on the recent estimates in the GBD analysis [[23](#_ENREF_23)]. In addition, we restricted the source of relative risks for tobacco smoking to the analysis from local large-scale cohorts in Taiwan, since we used the estimates of current smokers’ and never-smokers’ lung cancer mortality of NHIS and Civil Servant cohort to calculate SIR [[9](#_ENREF_9), [10](#_ENREF_10)].

For domestic risk factors (betel nut use, HBV infection, and HCV infection), we conducted systematic reviews of previous studies from the population of Taiwan. To be selected as a high-quality study from the population of Taiwan, we only included large prospective observational studies (cohort studies or population-based nested case-control studies) [[24-28](#_ENREF_24)]. If more than two studies were included in the systematic review, we pooled the effect estimates using a random-effects model. We used a null association (RR=1) if the included RRs were not statistically different from 1. See Appendix Tables 6-12 for the detailed RRs used in the analysis.

**Joint Effects of Multiple Risk Factors**

Since the cardiometabolic risk factors of high blood pressure, high blood glucose, high total cholesterol, and high BMI are often correlated with each other at the population level and the effects of BMI on CVDs have been shown to be mediated by other risk factors [[29](#_ENREF_29), [30](#_ENREF_30)], we estimated the joint effect of these cardiometabolic risk factors on CVDs, accounting for risk factor correlation and mediation effects. The correlation between risk factors was estimated from the individual records in the TW3H study. We used an additive excess risk scale to calculate the joint relative risks. According to previous meta-analyses, we adopted a 50% reduction of the excess risk of high BMI on cardiovascular deaths to account for the mediated effects through other risk factors (high blood pressure, high blood glucose, and high total cholesterol) [[29-31](#_ENREF_29)]. We summed the combined risks for individual records to compute PAF for the joint effects of these cardiovascular risks [[14](#_ENREF_14), [31](#_ENREF_31)]. The detailed results of the number of deaths attributable to joint cardiometabolic risk factors are presented in Appendix Figure 3.

**Redistribution of Garbage Codes**

To enhance comparability, we developed a multiple logistic regression model using Multiple Causes of Death data to generate a redistribution algorithm for garbage codes (GCs). Age, sex, counties, marriage status, death month, and the level of health care facility that issued the death certificate were selected as covariates in the model. For public health purposes, we do not assign a unique underlying cause of death for each death certified to GC. Instead, each death of GC can be distributed among several underlying cause of deaths, which reflects the uncertainty about the true underlying cause. Appendix Table 13 provides a listing of the number of each type of GC that we identified according to the WHO report for global causes of death, including septicemia, volume depletion, ill-defined cancer site, heart failure, ill-defined cardiovascular diseases, renal failure, ill-defined injury, ill-defined conditions, and other ill-defined codes. The largest category of GCs in Taiwan is ill-defined conditions, 30.4% among GCs. The algorithms for reassigning each of the GCs have been developed in R. After redistribution, new cause-specific death numbers and rates were calculated. The R code for the garbage code redistribution algorithm is available upon request to the authors.

**
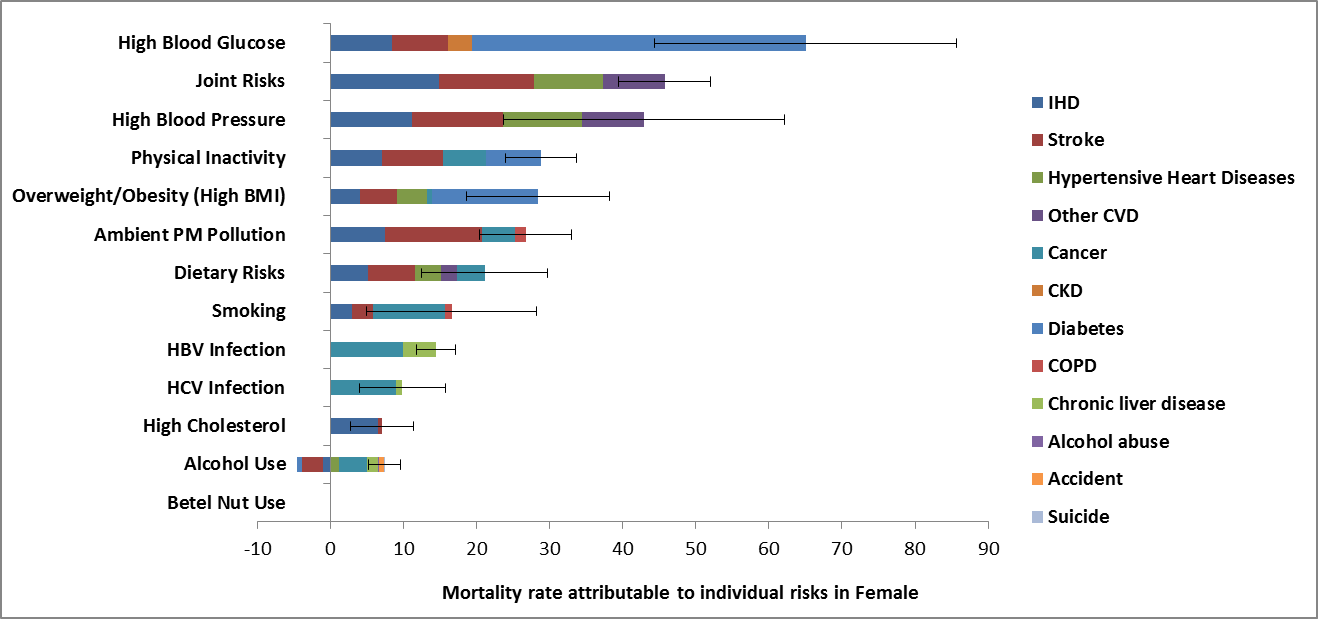

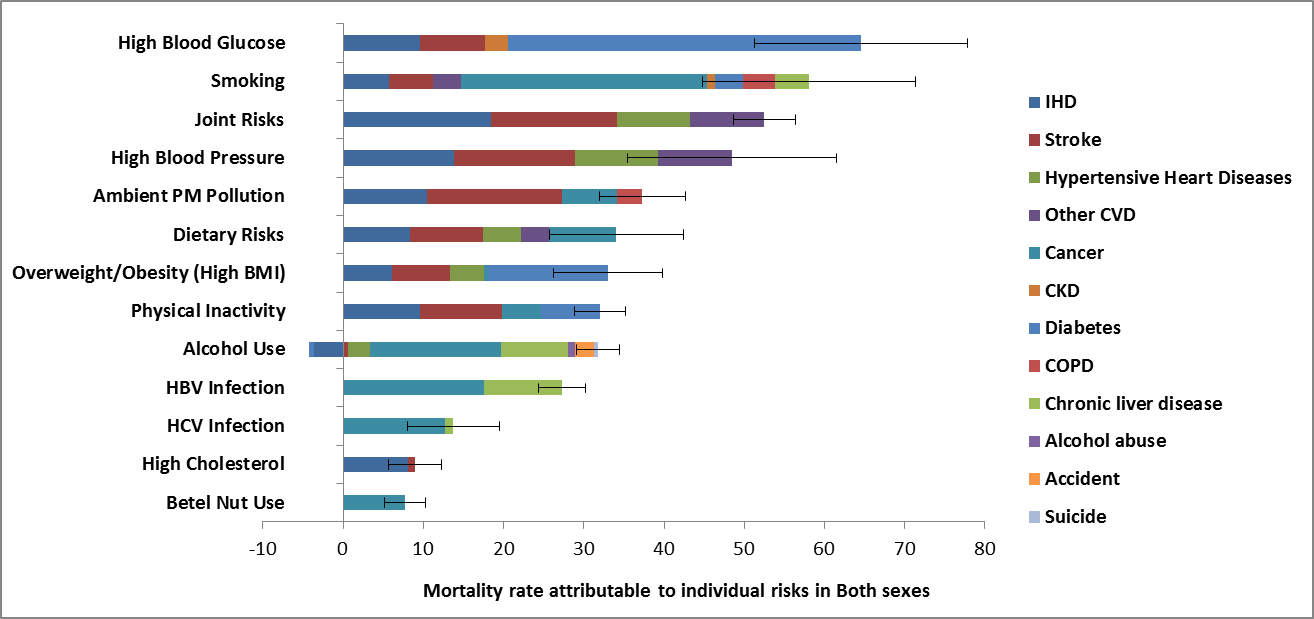

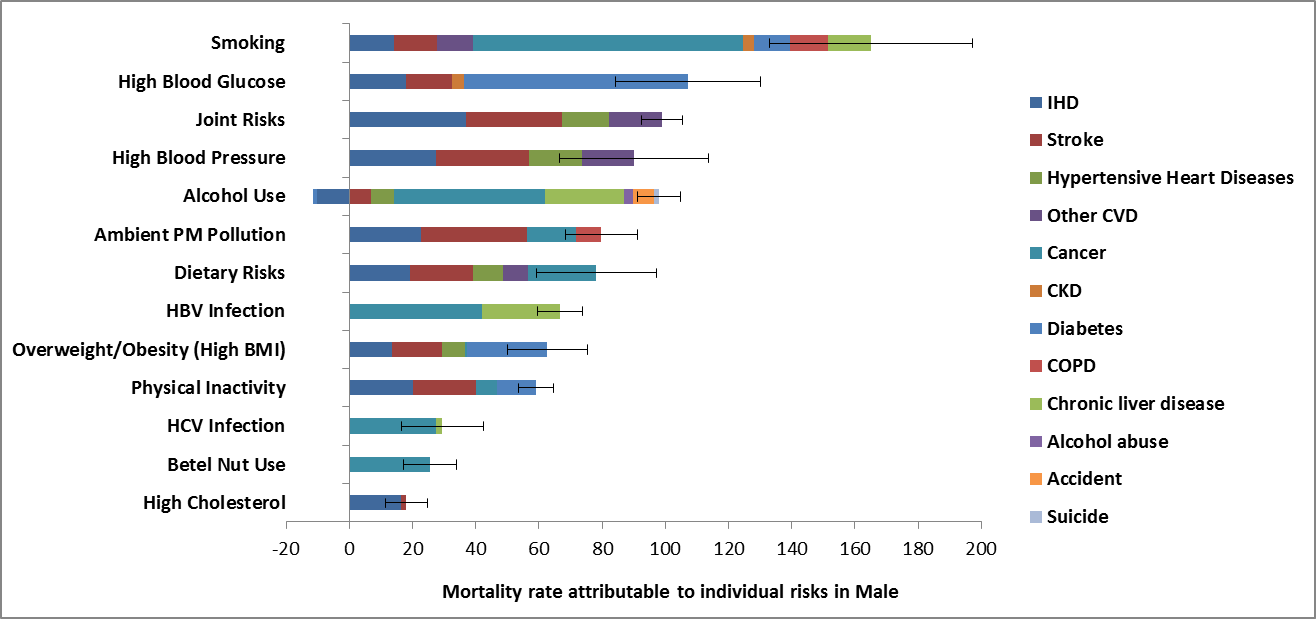
**

**Figure S1. Mortality rate (per 100,000 population) attributable to modifiable risk factors by cause** **in both sexes (A), in men (B), and in women (C)** IHD: ischemic heart disease; CVD: cardiovascular disease; CKD: chronic kidney disease; COPD: chronic obstructive pulmonary disease; BMI: body mass index

(C)

(B)

(A)

**
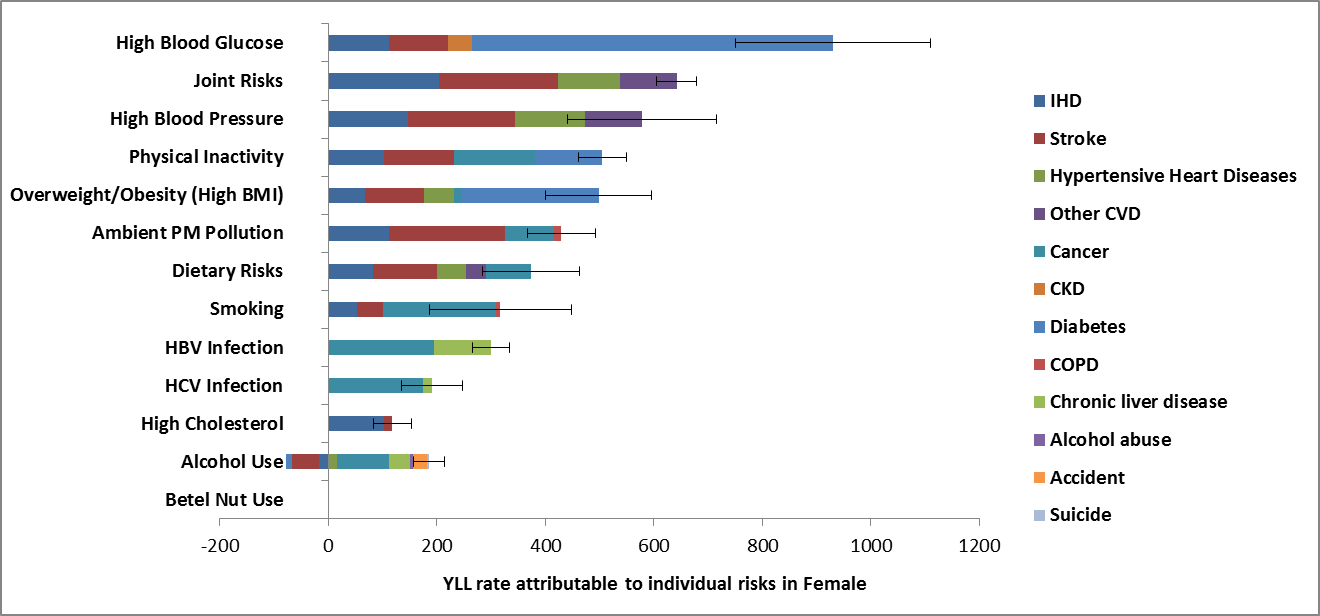

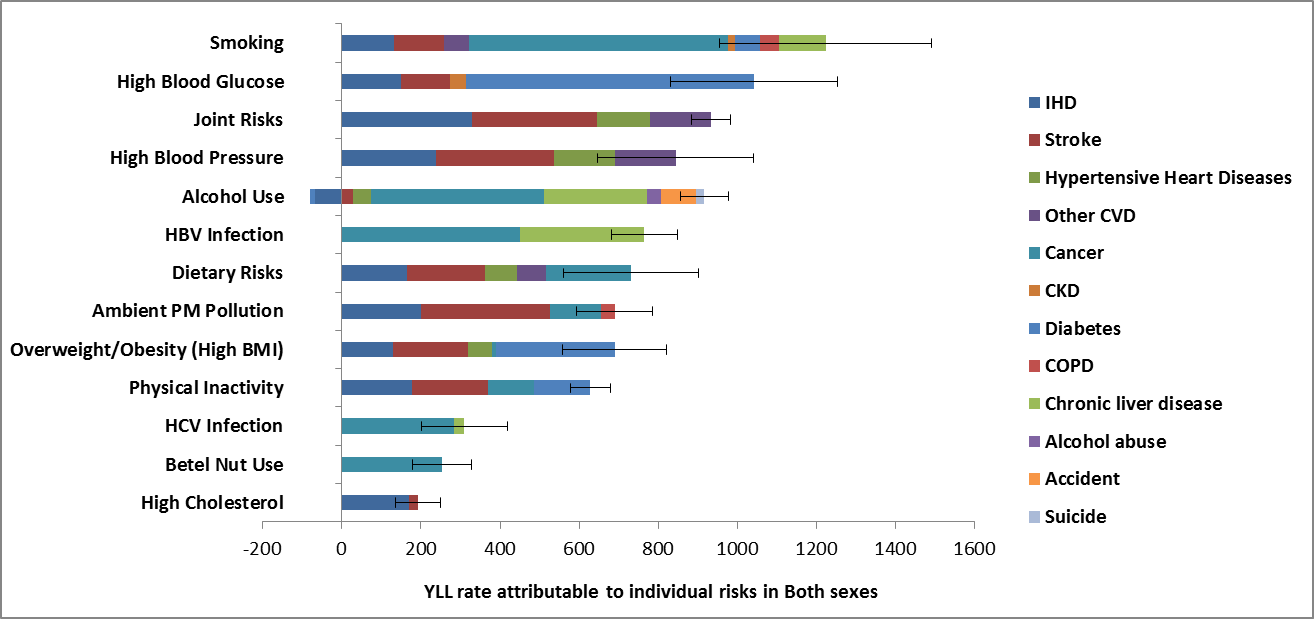

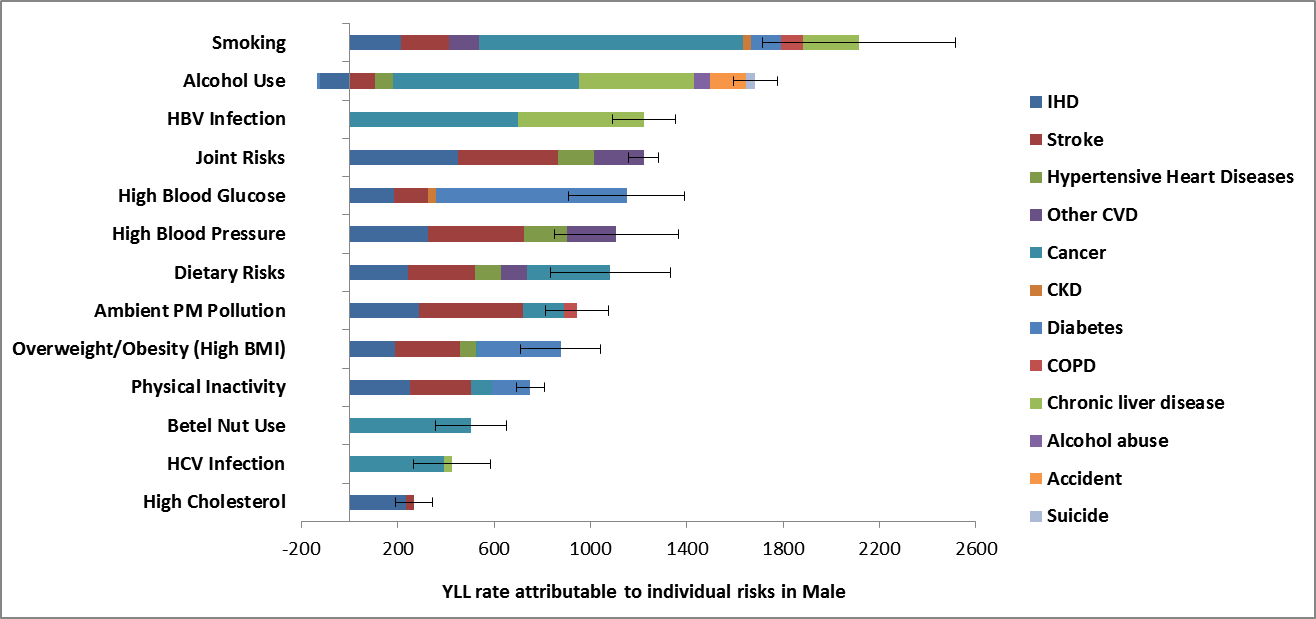
**

**Figure S2. Years of life lost rate (per 100,000 person-year) attributable to modifiable risk factors by cause** **in both sexes (A), in men (B), and in women (C)** IHD: ischemic heart disease; CVD: cardiovascular disease; CKD: chronic kidney disease; COPD: chronic obstructive pulmonary disease; BMI: body mass index

(C)

(B)

(A)


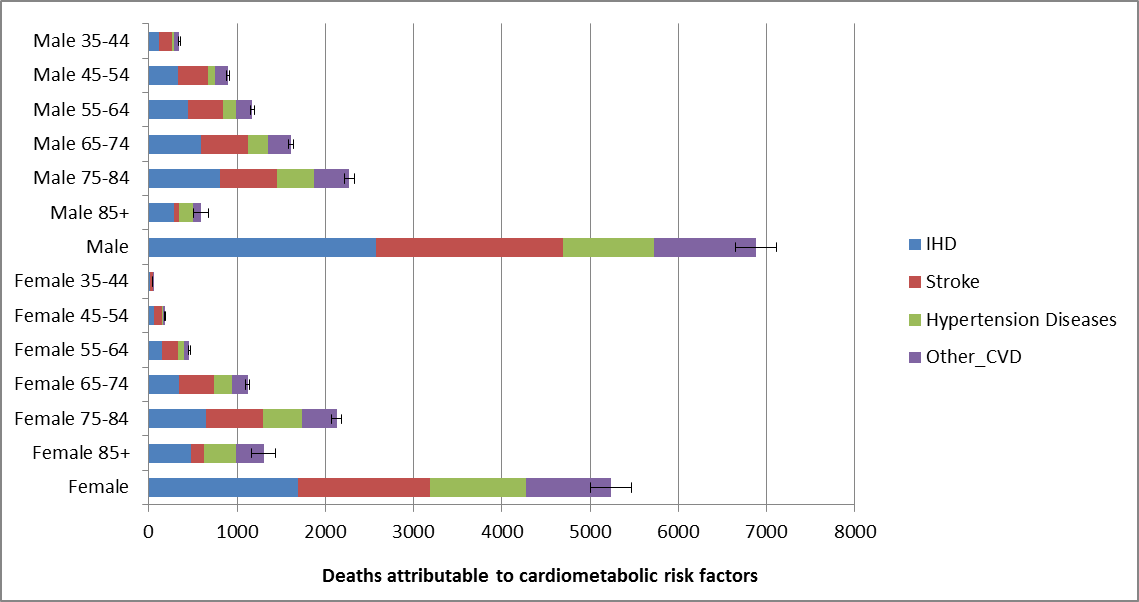


**Figure S3. Deaths attributable to cardiometabolic risk factors by age groups** **in both sexes** (cardiometabolic risk factor included high blood pressure, high blood glucose, high total cholesterol, and high BMI) IHD: ischemic heart disease; CVD: cardiovascular disease

**Table S1.**

| **Disease outcome** | **ICD-10** |
| --- | --- |
| **Ischemic heart disease** | I20-I25 |
| **Cerebrovascular disease** | I60-I69 |
| **Hypertensive heart disease** | I10-I13 |
| **Other cardiovascular disease** | I00, I26-I28, I34-I37, I44-I51, I70-I99 |
| **Diabetes** | E10-E14 |
| **Cancer** | C00-D48 |
| **COPD** | J40-J44, J47 |
| **Chronic liver disease & cirrhosis** | K70, K74 |
| **Chronic kidney disease** | N18 |
| **Alcohol abuse** | F10 |
| **Injury** | V00-V99 |
| **Suicide** | X71-X83 |

COPD: chronic obstructive pulmonary disease

| **TableS 2.** **Exposure to metabolic risk factors by sex and age group, 2009** | | | | | | | | | | | | | | | | | | |  | | |
| --- | --- | --- | --- | --- | --- | --- | --- | --- | --- | --- | --- | --- | --- | --- | --- | --- | --- | --- | --- | --- | --- |
| **Risk factor** | **Age group** | | | | | | | | | | | | | | | | | | | | |
|  | **35**–**44 years** | | | **45**–**54 years** | | | **55**–**64 years** | | | **65**–**74 years** | | | | **75**–**84 years** | | | **≥85 years** | | | | |
|  | **N** | **Mean** | **SE** | **N** | **Mean** | **SE** | **N** | **Mean** | **SE** | **N** | **Mean** | **SE** | **N** | | **Mean** | **SE** | | **N** | | **Mean** | **SE** |
| **Men** |  |  |  |  |  |  |  |  |  |  |  |  |  | |  |  | |  | |  |  |
| Systolic blood pressure (mm Hg) | 404 | 121.4 | 0.7 | 459 | 126.1 | 0.8 | 302 | 129.3 | 1.1 | 203 | 133.6 | 1.2 | 135 | | 132.9 | 1.4 | | 15 | | 122.3 | 6.2 |
| Total cholesterol (mmol/l) | 404 | 4.7 | 0.05 | 459 | 4.9 | 0.05 | 302 | 4.7 | 0.1 | 203 | 4.6 | 0.1 | 135 | | 4.4 | 0.1 | | 15 | | 4.4 | 0.3 |
| Fasting plasma glucose (mmol/l) | 404 | 5.1 | 0.1 | 459 | 5.6 | 0.2 | 302 | 5.6 | 0.1 | 203 | 6.0 | 0.4 | 135 | | 6.1 | 0.5 | | 15 | | 5.3 | 0.2 |
| Body mass index (kg/m^2^) | 1686 | 24.8 | 0.1 | 1643 | 24.8 | 0.1 | 962 | 24.8 | 0.1 | 581 | 23.9 | 0.1 | 406 | | 23.5 | 0.2 | | 83 | | 23.2 | 0.4 |
| **Women** |  |  |  |  |  |  |  |  |  |  |  |  |  | |  |  | |  | |  |  |
| Systolic blood pressure (mm Hg) | 471 | 110.8 | 0.6 | 586 | 119.5 | 0.8 | 380 | 127.8 | 0.9 | 241 | 135.1 | 1.2 | 113 | | 137.8 | 1.8 | | 24 | | 138.1 | 5.4 |
| Total cholesterol (mmol/l) | 471 | 4.5 | 0.04 | 586 | 5.0 | 0.04 | 380 | 5.1 | 0.1 | 241 | 5.1 | 0.1 | 113 | | 4.7 | 0.1 | | 24 | | 4.6 | 0.2 |
| Fasting plasma glucose (mmol/l) | 472 | 5.0 | 0.2 | 585 | 5.2 | 0.1 | 380 | 5.7 | 0.2 | 242 | 6.2 | 0.3 | 113 | | 6.8 | 0.8 | | 24 | | 5.6 | 0.3 |
| Body mass index (kg/m^2^) | 1836 | 22.4 | 0.1 | 1854 | 23.7 | 0.1 | 1252 | 24.6 | 0.1 | 805 | 24.4 | 0.1 | 400 | | 24.1 | 0.2 | | 97 | | 22.5 | 0.3 |

SE, standard error

| **Table S3.** **Exposure to lifestyle and infection risk factors by sex and age group, 2009** | | | | | | | | | | | |
| --- | --- | --- | --- | --- | --- | --- | --- | --- | --- | --- | --- |
| **Risk factor** | **Age group** | | | | | | | | | |  |
|  | **30**–**44 years** | | **45**–**59 years** | | **60**–**69 years** | | **70**–**79 years** | | **≥80 years** | |  |
|  | **N** | **%** | **N** | **%** | **N** | **%** | **N** | **%** | **N** | **%** |  |
| **Men** |  |  |  |  |  |  |  |  |  |  |  |
| Have intense physical activity (%) | 462 | 15.7 | 723 | 28.0 | 248 | 32.2 | 203 | 39.1 | 63 | 26.0 |  |
| Abstainers from alcohol (%) | 629 | 21.3 | 637 | 24.5 | 243 | 31.2 | 195 | 36.3 | 121 | 44.9 |  |
| Never or former betel nut users (%) | 2340 | 79.2 | 2173 | 83.8 | 718 | 92.7 | 513 | 95.7 | 265 | 98.5 |  |
| Smoking impact ratio (%) |  | 14.3 |  | 29.0 |  | 42.7 |  | 55.2 |  | 22.7^a^ |  |
| Hepatitis B infection* (%) | 202 | 21.4 | 137 | 17.7 | 31 | 10.9 | 18 | 5.7 | 2 | 1.4 |  |
| Hepatitis C infection** (%) | 28 | 3.0 | 30 | 4.0 | 19 | 6.1 | 14 | 6.0 | 1 | 2.5 |  |
| **Women** |  |  |  |  |  |  |  |  |  |  |  |
| Have intense physical activity (%) | 439 | 15.3 | 804 | 31.5 | 291 | 35.3 | 183 | 31.8 | 46 | 21.2 |  |
| Abstainer (%) | 1518 | 52.9 | 1582 | 61.7 | 648 | 77.7 | 506 | 83.6 | 221 | 85.4 |  |
| Never or former betel nut users (%) | 2839 | 99.0 | 2537 | 98.9 | 819 | 98.3 | 600 | 99 | 255 | 98.5 |  |
| Smoking impact ratio (%) |  | 5.6^a^ |  | 4.4^a^ |  | 3.5^a^ |  | 2.8^a^ |  | 4.3^a^ |  |
| Hepatitis B infection* (%) | 143 | 13.1 | 102 | 13.3 | 32 | 7.6 | 11 | 8.0 | 1 | 4.6 |  |
| Hepatitis C infection** (%) | 34 | 3.2 | 51 | 5.8 | 37 | 10.0 | 19 | 11.0 | 1 | 2.3 |  |

a: the prevalence of current smoking behavior

* Defined by the presence of HBsAg.

** Defined by the presence of anti-HCV antibody.

| **TableS 4. Annual average of PM_2.5_ exposure (μg/m^3^) by county, 2014** | | |
| --- | --- | --- |
| County | Mean | Standard error |
| Keelung | 21.3 | 0.16 |
| Taipei | 30.5 | 0.09 |
| New-Taipei | 29.2 | 0.09 |
| Taoyuan | 29.7 | 0.20 |
| Hsinchu | 31.0 | 0.21 |
| Yilan | 19.5 | 0.15 |
| Miaoli | 30.3 | 0.20 |
| Taichung | 38.7 | 0.25 |
| Jhanghua | 40.4 | 0.23 |
| Nantou | 36.6 | 0.22 |
| Yunlin | 43.7 | 0.25 |
| Chiayi | 46.0 | 0.27 |
| Tainan | 42.7 | 0.24 |
| Kaohsiung | 45.1 | 0.12 |
| Pingdong | 44.9 | 0.26 |
| Magong | 22.6 | 0.16 |
| Hualian | 22.0 | 0.12 |
| Taidong | 14.1 | 0.12 |
| Kinmen | 38.8 | 0.30 |
| Matsu | 29.6 | 0.23 |
| Taiwan | 33.0 | 0.03 |

| **Table S5. Number of selected risk-factor-attributable deaths in Taiwan NBD 2009, and in the 2010 estimates from GBD 2015, by sex** | | | | | | | | |
| --- | --- | --- | --- | --- | --- | --- | --- | --- |
|  | **Taiwan NBD 2009** | | |  | **GBD 2010*** | | |  |
| **Risk factor** | **Men** | **Women** | **Overall** |  | **Men** | **Women** | **Overall** |  |
|  |  |  |  |  |  |  |  |  |
| **High blood pressure** | 6,280 | 4,910 | 11,190 |  | 12,190 | 9,060 | 21,250 |  |
| **High Total cholesterol** | 1,260 | 810 | 2,070 |  | 5,410 | 3,640 | 9,050 |  |
| **High blood glucose** | 7,460 | 7,440 | 14,900 |  | 10,060 | 8,780 | 18,840 |  |
| **Overweight/obesity** | 4,360 | 3,250 | 7,610 |  | 5,900 | 4,300 | 10,200 |  |
| **Tobacco smoking** | 11,500 | 1,900 | 13,400 |  | 22,410 | 5,510 | 27,920 |  |
| **Alcohol use** | 6,030 | 320 | 6,350 |  | 10,270 | 1,600 | 11,870 |  |
| **Physical inactivity** | 4,100 | 3,300 | 7,400 |  | 2,640 | 1,070 | 3,710 |  |
| **High sodium intake** | 2,700 | 1,260 | 3,960 |  | 2,900 | 2,100 | 5,000 |  |
| **Low intake of fruit and vegetable** | 1,940 | 740 | 2,680 |  | 5,010 | 2,530 | 7,540 |  |
| **Air pollution** | 5,550 | 3,050 | 8,600 |  | 6,130 | 3,470 | 9,600 |  |
| **Betel nut use** | 1,780 | 0 | 1,780 |  | -- | -- | -- |  |
| **HBV infection** | 4,650 | 1,650 | 6,300 |  | -- | -- | -- |  |
| **HCV infection** | 2,050 | 1,120 | 3,170 |  | -- | -- | -- |  |
| *Estimates were obtained from the GBD 2015 project | | | | | | | | |

**TableS 6. Relative risks for the effects of metabolic risk factors on disease outcomes**

| **Risk factor, disease** | **Sex** | **Age (years)** | |  |  |  |  | **Reference** |
| --- | --- | --- | --- | --- | --- | --- | --- | --- |
|  |  | **35**–**44** | **45**–**54** | **55**–**64** | **65**–**74** | **75**–**84** | **≥85** |  |
| ***High blood pressure (per 10 mmHg increase)*** |  |  |  |  |  |  |  |  |
| Ischemic heart disease | Both | 1.68 | 1.56 | 1.45 | 1.33 | 1.26 | 1.14 | [[16](#_ENREF_16)] |
| Ischemic stroke | Both | 2.05 | 1.83 | 1.63 | 1.44 | 1.28 | 1.10 | [[16](#_ENREF_16)] |
| Hemorrhage stroke | Both | 2.11 | 1.89 | 1.66 | 1.46 | 1.29 | 1.10 ^a^ | [[16](#_ENREF_16)] |
| Hypertensive heart disease | Both | 2.86 | 2.49 | 2.16 | 1.88 | 1.63 | 1.37 | [[16](#_ENREF_16)] |
| Other CVD | Both | 1.44 | 1.37 | 1.30 | 1.24 | 1.18 | 1.11 | [[16](#_ENREF_16)] |
| ***High total cholesterol (per mmol/L increase)*** |  |  |  |  |  |  |  |  |
| Ischemic heart disease | Both | 2.20 | 1.82 | 1.44 | 1.27 | 1.18 | 1.30 | [[16](#_ENREF_16)] |
| Ischemic stroke | Both | 1.71 | 1.41 | 1.20 | 1.08 | 1.03 ^a^ | 0.92 ^a^ | [[16](#_ENREF_16)] |
| ***High blood glucose (per mmol/L increase)*** |  |  |  |  |  |  |  |  |
| Ischemic heart disease | Both | 1.21 | 1.19 | 1.18 | 1.16 | 1.16 | 1.14 | [[16](#_ENREF_16)] |
| Total stroke | Both | 1.19 ^a^ | 1.16 ^a^ | 1.14 | 1.14 | 1.10 | 1.06 ^a^ | [[16](#_ENREF_16)] |
| CKD | Both | 1.26 | 1.26 | 1.26 | 1.26 | 1.26 | 1.26 | [[17](#_ENREF_17)] |
| ***High body mass index (per 5 kg/m^2^ increase)*** |  |  |  |  |  |  |  |  |
| Ischemic heart disease | Both | 1.66 | 1.55 | 1.44 | 1.35 | 1.26 | 1.14 | [[16](#_ENREF_16)] |
| Ischemic stroke | Both | 1.86 | 1.67 | 1.50 | 1.35 | 1.21 | 1.04 ^a^ | [[16](#_ENREF_16)] |
| Hemorrhage stroke | Both | 2.54 | 2.10 | 1.75 | 1.48 | 1.30 | 1.05 ^a^ | [[16](#_ENREF_16)] |
| Hypertensive heart disease | Both | 2.15 ^a^ | 2.02^a^ | 1.90 | 1.81 | 1.63 | 1.45 | [[16](#_ENREF_16)] |
| Colon cancer | Both | 1.03 | 1.03 | 1.03 | 1.03 | 1.03 | 1.03 | [[18](#_ENREF_18)] |
| Kidney cancer | Both | 1.06 | 1.06 | 1.06 | 1.06 | 1.06 | 1.06 | [[18](#_ENREF_18)] |
| Breast cancer | Female | 1.03 | 1.03 | 1.03 | 1.03 | 1.03 | 1.03 | [[18](#_ENREF_18)] |
| Diabetes mellitus | Both | 3.07 | 2.66 | 2.32 | 2.03 | 1.70 | 1.38 | [[16](#_ENREF_16)] |

CVD: cardiovascular disease; CKD: chronic kidney disease

^a^ We replaced these statistically insignificant relative risks with 1 in our analysis.

**Table S7. Relative risks for the effects of dietary risks on disease outcomes.**

| **Dietary risks** | **Disease outcome** | **Age (years) ^b^** | **RR** | **Reference** |
| --- | --- | --- | --- | --- |
| High sodium intake  (per 100 mmol/d increase) | Systolic blood pressure (SBP) (mmHg) | SBP**≥**140 mmHg | 7.11 | [[19](#_ENREF_19)] |
|  |  | SBP**<**140 mmHg | 3.57 | [[19](#_ENREF_19)] |
| Low intake of fruits  and vegetables  (per 80 g/d lower intake) | Ischemic heart disease | 30–69 | 1.04 | [[32](#_ENREF_32)] |
|  |  | 70–79 | 1.03 | [[32](#_ENREF_32)] |
|  |  | ≥80 | 1.02 | [[32](#_ENREF_32)] |
|  | Ischemic stroke | 30–69 | 1.06 | [[33](#_ENREF_33)] |
|  |  | 70–79 | 1.05 | [[33](#_ENREF_33)] |
|  |  | ≥80 | 1.03 | [[33](#_ENREF_33)] |
|  | Lung cancer | 30–69 | 1.04 | [[34](#_ENREF_34)] |
|  |  | 70–79 | 1.03 | [[34](#_ENREF_34)] |
|  |  | ≥80 | 1.02 | [[34](#_ENREF_34)] |
|  | Stomach cancer | 30–69 | 1.06 | [[34](#_ENREF_34)] |
|  |  | 70–79 | 1.05 | [[34](#_ENREF_34)] |
|  |  | ≥80 | 1.03 | [[34](#_ENREF_34)] |
|  | Colorectal cancer | 30–69 | 1.01 | [[34](#_ENREF_34)] |
|  |  | 70–79 | 1.01 | [[34](#_ENREF_34)] |
|  |  | ≥80 | 1.00 | [[34](#_ENREF_34)] |
|  | Esophagus, mouth,  and pharynx cancers | 30–69 | 1.10 | [[35](#_ENREF_35)] |
|  |  | 70–79 | 1.08 | [[35](#_ENREF_35)] |
|  |  | ≥80 | 1.05 | [[35](#_ENREF_35)] |

**TableS 8. Relative risks for the effects of physical inactivity on disease outcomes.**

| **Disease outcome** | **Age (years) ^b^** | **I ^c^** | **II** | **III** |
| --- | --- | --- | --- | --- |
| Ischemic heart disease | 30–69 | 1.00 | 1.44 | 1.71 |
|  | 70–79 | 1.00 | 1.31 | 1.50 |
|  | ≥80 | 1.00 | 1.20 | 1.30 |
| Ischemic stroke | 30–69 | 1.00 | 1.10 ^a^ | 1.53 |
|  | 70–79 | 1.00 | 1.08 ^a^ | 1.38 |
|  | ≥80 | 1.00 | 1.05 ^a^ | 1.24 |
| Breast cancer | 30–44 | 1.00 | 1.13 | 1.25 |
|  | 45–69 | 1.00 | 1.13 | 1.34 |
|  | 70–79 | 1.00 | 1.09 | 1.25 |
|  | ≥80 | 1.00 | 1.06 ^a^ | 1.16 |
| Colon cancer | 30–69 | 1.00 | 1.18 | 1.68 |
|  | 70–79 | 1.00 | 1.13 | 1.48 |
|  | ≥80 | 1.00 | 1.08 ^a^ | 1.30 |
| Diabetes mellitus | 30–69 | 1.00 | 1.24 | 1.45 |
|  | 70–79 | 1.00 | 1.18 | 1.32 |
|  | ≥80 | 1.00 | 1.11 ^a^ | 1.20 |

We obtained all relative risks from Bull et al. (2004) [[4](#_ENREF_4)]

^a^ We replaced these statistically insignificant relative risks with 1 in our analysis.

^b^ Except for breast cancer, relative risks were estimated for both sexes combined.

^c^ Bull et al. (2004) originally defined the categories as: I, “≥2.5 hr/wk of moderate activity or ≥1 hr/wk of vigorous activity”; II, “<2.5h/wk of moderate activity or < 1h/wk of vigorous activity”; III, “no moderate or vigorous activity”. We used self-reports on the intensity of physical activity and substituted “highly intense,” “moderately intense,” and “poorly intense” for I, II, and III, respectively.

**TableS 9. Relative risks for the effects of tobacco smoking (active smoking) on disease outcomes**

| **Risk factor, disease** | **Sex** | **Age (years)** |  |  |  |  | **Reference** |
| --- | --- | --- | --- | --- | --- | --- | --- |
| ***Tobacco smoking*** |  | **35**–**44** | **45**–**59** | **60**–**69** | **70**–**79** | **≥80** |  |
| Ischemic heart disease | Men | 2.06 | 2.06 | 2.06 | 1.00 | 1.00 | [[9](#_ENREF_9), [10](#_ENREF_10)] |
|  | Women | 3.58 | 3.58 | 3.58 | 1.00 | 1.00 | [[9](#_ENREF_9), [10](#_ENREF_10)] |
| Total stroke | Men | 1.65 | 1.65 | 1.65 | 1.00 | 1.00 | [[9](#_ENREF_9), [10](#_ENREF_10)] |
|  | Women | 2.08 | 2.08 | 2.08 | 1.00 | 1.00 | [[9](#_ENREF_9), [10](#_ENREF_10)] |
| Other CVD | Men | 1.60 | 1.96 | 1.96 | 1.96 | 1.96 | [[9](#_ENREF_9), [10](#_ENREF_10)] |
| Diabetes mellitus | Men | 1.51 | 1.51 | 1.51 | 1.51 | 1.51 | [[9](#_ENREF_9), [10](#_ENREF_10)] |
|  | Women | 1.09 ^a^ | 1.09 ^a^ | 1.09 ^a^ | 1.09 ^a^ | 1.09 ^a^ | [[9](#_ENREF_9), [10](#_ENREF_10)] |
| Oral cancer | Men | 2.60 | 2.60 | 2.60 | 2.60 | 2.60 | [[9](#_ENREF_9), [10](#_ENREF_10)] |
| Nasopharyngeal cancer | Men | 1.78 | 1.78 | 1.78 | 1.78 | 1.78 | [[9](#_ENREF_9), [10](#_ENREF_10)] |
| Esophageal cancer | Men | 3.18 | 3.18 | 3.18 | 3.18 | 3.18 | [[9](#_ENREF_9), [10](#_ENREF_10)] |
| Stomach cancer | Men | 1.68 | 1.68 | 1.68 | 1.68 | 1.68 | [[9](#_ENREF_9), [10](#_ENREF_10)] |
| Rectal cancer | Men | 2.06 | 2.06 | 2.06 | 2.06 | 2.06 | [[9](#_ENREF_9), [10](#_ENREF_10)] |
| Liver cancer | Men | 1.46 | 1.46 | 1.46 | 1.46 | 1.46 | [[9](#_ENREF_9), [10](#_ENREF_10)] |
|  | Women | 5.03 | 5.03 | 5.03 | 5.03 | 5.03 | [[9](#_ENREF_9), [10](#_ENREF_10)] |
|  |  |  |  |  |  |  |  |
|  |  |  |  |  |  |  |  |
| Lung cancer | Men | 2.73 | 2.73 | 2.73 | 2.73 | 2.73 | [[9](#_ENREF_9), [10](#_ENREF_10)] |
|  | Women | 3.36 | 3.36 | 3.36 | 3.36 | 3.36 | [[9](#_ENREF_9), [10](#_ENREF_10)] |
| Cervical cancer | Women | 5.78 | 5.78 | 5.78 | 5.78 | 5.78 | [[9](#_ENREF_9), [10](#_ENREF_10)] |
| Chronic liver disease | Men | 2.01 | 2.01 | 2.01 | 2.01 | 2.01 | [[9](#_ENREF_9), [10](#_ENREF_10)] |
|  | Women | 0.71 ^a^ | 0.71 ^a^ | 0.71 ^a^ | 0.71 ^a^ | 0.71 ^a^ | [[9](#_ENREF_9), [10](#_ENREF_10)] |
| CKD | Men | 2.23 | 2.23 | 2.23 | 2.23 | 2.23 | [[9](#_ENREF_9), [10](#_ENREF_10)] |
|  | Women | 0.94 ^a^ | 0.94 ^a^ | 0.94 ^a^ | 0.94 ^a^ | 0.94 ^a^ | [[9](#_ENREF_9), [10](#_ENREF_10)] |
| COPD | Men | 1.82 | 1.82 | 1.82 | 1.82 | 1.82 | [[9](#_ENREF_9), [10](#_ENREF_10)] |
|  | Women | 2.87 | 2.87 | 2.87 | 2.87 | 2.87 | [[9](#_ENREF_9), [10](#_ENREF_10)] |

CVD: cardiovascular disease, CKD: chronic kidney disease, COPD: chronic obstructive pulmonary disease

^a^ We replaced these statistically insignificant relative risks with 1 in our analysis.

**Table S10. Relative risks for the effects of tobacco smoking (passive smoking) on disease outcomes**

| **Risk factor, disease ^a^** | **Sex** | **RR** | **Reference** |
| --- | --- | --- | --- |
| ***Passive smoking*** |  |  |  |
| Ischemic heart disease | Both | 1.27 | [[12](#_ENREF_12)] |
| Total stroke | Both | 1.22 | [[12](#_ENREF_12)] |
| Lung cancer | Both | 1.43 | [[12](#_ENREF_12)] |

CVD: cardiovascular disease

^a^ For these risk factor–disease pairs, RRs in the source were reported for all ages combined.

**Table S11. Relative risks for the effects of alcohol use on disease outcomes.**

| **Disease outcome^a^** | **Sex** | **Abstainers** | **DI^b^** | **DII** | **DIII** |
| --- | --- | --- | --- | --- | --- |
| Ischemic heart disease | Men | 1.00 | 0.82 | 0.83 | 1.00 |
|  | Women | 1.00 | 0.82 | 0.83 | 1.12 |
| Ischemic stroke | Men | 1.00 | 0.94 | 1.33 | 1.65 |
|  | Women | 1.00 | 0.52 | 0.64 | 1.06 |
| Hemorrhage stroke | Men | 1.00 | 1.27 | 2.19 | 2.38 |
|  | Women | 1.00 | 0.59 | 0.64 | 1.06 |
| Hypertensive heart disease | Men | 1.00 | 1.40 | 2.00 | 4.10 |
|  | Women | 1.00 | 1.40 | 2.00 | 2.00 |
| Diabetes mellitus | Men | 1.00 | 1.00 | 0.57 | 0.73 |
|  | Women | 1.00 | 0.92 | 0.87 | 1.13 |
| Oral cancer | Both | 1.00 | 1.45 | 1.85 | 5.39 |
| Esophagus cancer | Both | 1.00 | 1.80 | 2.38 | 4.36 |
| Breast cancer | Women | 1.00 | 1.14 | 1.41 | 1.59 |
| Liver cancer | Both | 1.00 | 1.45 | 3.03 | 3.60 |
| Other cancer | Both | 1.00 | 1.10 | 1.30 | 1.70 |
| Chronic liver disease | Both | 1.00 | 1.30 | 9.50 | 13.00 |
| Traffic accident | Both | 1.00 | 1.20 | 1.70 | 4.00 |
| Suicide | Both | 1.00 | 1.00 | 1.00 | 2.10 |

We obtained all relative risks from Rehm et al. (2004) [[36](#_ENREF_36)]

^a^ For these risk factor–disease pairs, RRs in the source were reported for all ages combined.

^b^ Exposure categories were Abstainer, a person not having had a drink containing alcohol within the last year; DI, 0–19.99 g of pure alcohol daily (females) and 0–39.99 g (males); DII, 20–39.99 g (females) and 40–59.99 g (males); and DIII, ≥40 g (females) and ≥60 g (males).

**Table S12. Relative risks for the effects of domestic risk factors on disease outcomes.**

| **Risk factor, disease** | **Sex** | **RR** | **Reference** |
| --- | --- | --- | --- |
| ***Betel nut use*** |  |  |  |
| Oral cancer | Men | 12.52 | [[37](#_ENREF_37)] |
| Larynx cancer | Men | 6.24 | [[37](#_ENREF_37)] |
| Esophageal cancer | Men | 5.64 | [[37](#_ENREF_37)] |
| ***HBV infection*** |  |  |  |
| Liver cancer | Both | 11.61 | [[25-28](#_ENREF_25)] |
| Chronic liver disease | Both | 6.63 | [[24](#_ENREF_24)] |
| ***HCV infection*** |  |  |  |
| Liver cancer | Both | 11.28 | [[26-28](#_ENREF_26)] |
| Chronic liver disease | Both | 1.80 | [[24](#_ENREF_24)] |

**Table S13. List of garbage codes with associated ICD-10 codes**

| **Code** | **Code name** | **ICD-10 code** | **N** | **(%)** |
| --- | --- | --- | --- | --- |
| GC_01 | Septicemia | A40, A41 | 3,623 | (16.0%) |
| GC_02 | Volume depletion | E86 | 6 | (0.03%) |
| GC_03 | Ill-defined cancer | C76, C80, C97 | 1,127 | (5.0%) |
| GC_04 | Heart failure | I50 | 3,174 | (14.0%) |
| GC_05 | Ill-defined cardiovascular diseases | I46, I472, I490, I514, I515, I516, I519, I709 | 1,889 | (8.3%) |
| GC_06 | Renal failure | N00-N17, N19, N25-N29 | 2,713 | (12.0%) |
| GC_07 | Ill-defined injury | Y10-Y34, Y872 | 557 | (2.5%) |
| GC_08 | Ill-defined conditions | R00-R99 | 6,880 | (30.4%) |
| GC_09 | Other ill-defined | D65, I99, J81, J96, I269, P285 | 2,690 | (11.9%) |

**Reference**

1. World Health Report. Reducing risks, promoting healthy life. Geneva: World Health Organization; 2002.

2. Ainsworth BE, Haskell WL, Herrmann SD, Meckes N, Bassett DR, Jr., Tudor-Locke C, Greer JL, Vezina J, Whitt-Glover MC, Leon AS: 2011 Compendium of Physical Activities: a second update of codes and MET values. Med Sci Sports Exerc 2011, 43:1575-1581.

3. Ainsworth BE, Haskell WL, Whitt MC, Irwin ML, Swartz AM, Strath SJ, O'Brien WL, Bassett DR, Jr., Schmitz KH, Emplaincourt PO, et al: Compendium of physical activities: an update of activity codes and MET intensities. Med Sci Sports Exerc 2000, 32:S498-504.

4. Bull FC AT, Dixon T, Ham S, Neiman A, et al. : Physical inactivity. In Comparative quantification of health risks: global and regional burden of disease attributable to selected major risk factors (Ezzati M LA, Rodgers A, Murray CJL ed. pp. 729-881. World Health Organization: Geneva; 2004:729-881.

5. Ezzati M, Henley SJ, Thun MJ, Lopez AD: Role of smoking in global and regional cardiovascular mortality. Circulation 2005, 112:489-497.

6. Peto R, Lopez AD, Boreham J, Thun M, Heath C, Jr.: Mortality from tobacco in developed countries: indirect estimation from national vital statistics. Lancet 1992, 339:1268-1278.

7. National Health Interview Survey. [<http://nhis.nhri.org.tw/2001nhis.html> (in Chinese)]

8. National Health Interview Survey. [<http://nhis.nhri.org.tw/2005nhis.html> (in Chinese)]

9. Wen C-P, Tsai SP, Chen C-J, Cheng T-Y: The mortality risks of smokers in Taiwan: Part I: cause-specific mortality. Preventive medicine 2004, 39:528-535.

10. Wen CP, Tsai SP, Chen CJ, Cheng TY, Tsai MC, Levy DT: Smoking attributable mortality for Taiwan and its projection to 2020 under different smoking scenarios. Tob Control 2005, 14 Suppl 1:i76-80.

11. Corrao G, Rubbiati L, Bagnardi V, Zambon A, Poikolainen K: Alcohol and coronary heart disease: a meta-analysis. Addiction 2000, 95:1505-1523.

12. Lim SS, Vos T, Flaxman AD, Danaei G, Shibuya K, Adair-Rohani H, Amann M, Anderson HR, Andrews KG, Aryee M, et al: A comparative risk assessment of burden of disease and injury attributable to 67 risk factors and risk factor clusters in 21 regions, 1990-2010: a systematic analysis for the Global Burden of Disease Study 2010. Lancet 2012, 380:2224-2260.

13. Lo WC, Shie RH, Chan CC, Lin HH: Burden of disease attributable to ambient fine particulate matter exposure in Taiwan. J Formos Med Assoc 2017, 116:32-40.

14. Ikeda N, Inoue M, Iso H, Ikeda S, Satoh T, Noda M, Mizoue T, Imano H, Saito E, Katanoda K, et al: Adult mortality attributable to preventable risk factors for non-communicable diseases and injuries in Japan: a comparative risk assessment. PLoS Med 2012, 9:e1001160.

15. Puddey IB, Rakic V, Dimmitt SB, Beilin LJ: Influence of pattern of drinking on cardiovascular disease and cardiovascular risk factors--a review. Addiction 1999, 94:649-663.

16. Singh GM, Danaei G, Farzadfar F, Stevens GA, Woodward M, Wormser D, Kaptoge S, Whitlock G, Qiao Q, Lewington S, et al: The age-specific quantitative effects of metabolic risk factors on cardiovascular diseases and diabetes: a pooled analysis. PLoS One 2013, 8:e65174.

17. Intensive blood-glucose control with sulphonylureas or insulin compared with conventional treatment and risk of complications in patients with type 2 diabetes (UKPDS 33). UK Prospective Diabetes Study (UKPDS) Group. Lancet 1998, 352:837-853.

18. W. Philip T. James RJ-L, Cliona Ni Mhurchu, Eleni Kalamara, Maryam Shayeghi, Neville J. Rigby, Chizuru Nishida and Anthony Rodgers: Overweight and obesity. In Comparative quantification of health risks: Global and regional burden of disease attributable to selected major risk factors (Ezzati M LA, Rogers A, Murray CJL, ed. pp. 497-596. World Health Organization: Geneva; 2004:497-596.

19. Danaei G, Ding EL, Mozaffarian D, Taylor B, Rehm J, Murray CJ, Ezzati M: The preventable causes of death in the United States: comparative risk assessment of dietary, lifestyle, and metabolic risk factors. PLoS Med 2009, 6:e1000058.

20. Willi C, Bodenmann P, Ghali WA, Faris PD, Cornuz J: Active smoking and the risk of type 2 diabetes: a systematic review and meta-analysis. JAMA 2007, 298:2654-2664.

21. Ezzati M, Henley SJ, Lopez AD, Thun MJ: Role of smoking in global and regional cancer epidemiology: current patterns and data needs. Int J Cancer 2005, 116:963-971.

22. Thun MJ, Apicella LF, Henley SJ: Smoking vs other risk factors as the cause of smoking-attributable deaths: confounding in the courtroom. JAMA 2000, 284:706-712.

23. Burnett RT, Pope CA, 3rd, Ezzati M, Olives C, Lim SS, Mehta S, Shin HH, Singh G, Hubbell B, Brauer M, et al: An integrated risk function for estimating the global burden of disease attributable to ambient fine particulate matter exposure. Environ Health Perspect 2014, 122:397-403.

24. Chen TH, Chen CJ, Yen MF, Lu SN, Sun CA, Huang GT, Yang PM, Lee HS, Duffy SW: Ultrasound screening and risk factors for death from hepatocellular carcinoma in a high risk group in Taiwan. Int J Cancer 2002, 98:257-261.

25. Fwu CW, Chien YC, Nelson KE, Kirk GD, You SL, Kuo HS, Feinleib M, Chen CJ: Mortality after chronic hepatitis B virus infection: a linkage study involving 2 million parous women from Taiwan. J Infect Dis 2010, 201:1016-1023.

26. Lai MS, Hsieh MS, Chiu YH, Chen TH: Type 2 diabetes and hepatocellular carcinoma: A cohort study in high prevalence area of hepatitis virus infection. Hepatology 2006, 43:1295-1302.

27. Wang CS, Yao WJ, Chang TT, Wang ST, Chou P: The impact of type 2 diabetes on the development of hepatocellular carcinoma in different viral hepatitis statuses. Cancer Epidemiol Biomarkers Prev 2009, 18:2054-2060.

28. Wang LY, You SL, Lu SN, Ho HC, Wu MH, Sun CA, Yang HI, Chien-Jen C: Risk of hepatocellular carcinoma and habits of alcohol drinking, betel quid chewing and cigarette smoking: a cohort of 2416 HBsAg-seropositive and 9421 HBsAg-seronegative male residents in Taiwan. Cancer Causes Control 2003, 14:241-250.

29. Lu Y, Hajifathalian K, Ezzati M, Woodward M, Rimm EB, Danaei G, C GBMRF: Metabolic mediators of the effects of body-mass index, overweight, and obesity on coronary heart disease and stroke: a pooled analysis of 97 prospective cohorts with 1.8 million participants. Lancet 2014, 383:970-983.

30. Lu Y, Hajifathalian K, Rimm EB, Ezzati M, Danaei G: Mediators of the Effect of Body Mass Index on Coronary Heart Disease Decomposing Direct and Indirect Effects. Epidemiology 2015, 26:153-162.

31. Danaei G, Rimm EB, Oza S, Kulkarni SC, Murray CJ, Ezzati M: The promise of prevention: the effects of four preventable risk factors on national life expectancy and life expectancy disparities by race and county in the United States. PLoS Med 2010, 7:e1000248.

32. Dauchet L, Amouyel P, Hercberg S, Dallongeville J: Fruit and vegetable consumption and risk of coronary heart disease: a meta-analysis of cohort studies. J Nutr 2006, 136:2588-2593.

33. Dauchet L, Amouyel P, Dallongeville J: Fruit and vegetable consumption and risk of stroke: a meta-analysis of cohort studies. Neurology 2005, 65:1193-1197.

34. Lock K PJ, Causer L, McKee M: Low fruit and vegetable consumption. In Comparative quantification of health risks: Global and regional burden of disease attributable to selected major risk factors (Ezzati M LA, Rogers A, Murray CJL, ed. pp. 579-728. World Health Organization: Geneva; 2004:579-728.

35. Boeing H, Dietrich T, Hoffmann K, Pischon T, Ferrari P, Lahmann PH, Boutron-Ruault MC, Clavel-Chapelon F, Allen N, Key T, et al: Intake of fruits and vegetables and risk of cancer of the upper aero-digestive tract: the prospective EPIC-study. Cancer Causes Control 2006, 17:957-969.

36. Rehm J RR, Monteiro M, Gmel G, Graham K, et al.: Alcohol use. In Comparative quantification of health risks: Global and regional burden of disease attributable to selected major risk factors (Ezzati M LA, Rogers A, Murray CJL, ed. pp. 959-1108. World Health Organization: Geneva; 2004:959-1108.

37. Wen CP, Tsai MK, Chung WS, Hsu HL, Chang YC, Chan HT, Chiang PH, Cheng TY, Tsai SP: Cancer risks from betel quid chewing beyond oral cancer: a multiple-site carcinogen when acting with smoking. Cancer Causes Control 2010, 21:1427-1435.
